# Biologically Active Compounds in True Slime Molds and Their Prospects for Sustainable Pest and Pathogen Control

**DOI:** 10.3390/ijms26051951

**Published:** 2025-02-24

**Authors:** Tomasz Pawłowicz, Konrad Wilamowski, Monika Puchlik, Igor Żebrowski, Gabriel Michał Micewicz, Karolina Anna Gabrysiak, Piotr Borowik, Tadeusz Malewski, Ewa Zapora, Marek Wołkowycki, Tomasz Oszako

**Affiliations:** 1 Institute of Forest Sciences, Faculty of Civil Engineering and Environmental Sciences, Białystok University of Technology, ul. Wiejska 45E, 15-351 Białystok, Poland; k.wilamowski@pb.edu.pl (K.W.); m.puchlik@pb.edu.pl (M.P.); 86774@student.pb.edu.pl (I.Ż.); 87027@student.pb.edu.pl (G.M.M.); karolina.gabrysiak@pb.edu.pl (K.A.G.); e.zapora@pb.edu.pl (E.Z.); wolkm@poczta.onet.pl (M.W.); 2 Forest Protection Department, Forest Research Institute in Sękocin Stary, ul. Braci Leśnej 3, 05-090 Raszyn, Poland; pborow@poczta.onet.pl; 3 Department of Molecular and Biometric Techniques, Museum and Institute of Zoology, 00-818 Warsaw, Poland; tmalewski@miiz.waw.pl

**Keywords:** true slime molds, bioactive compounds, secondary metabolites, developmental stages, pest management, pathogen control

## Abstract

True slime molds (*Eumycetozoa*) represent a monophyletic clade within the phylum *Amoebozoa*, comprising the lineages *Myxogastria*, *Dictyostelia*, and *Protosporangiida*. Although historically misclassified as fungi, recent molecular and biochemical studies underscore their distinct evolutionary trajectories and rich metabolomic profiles. In this review, we synthesize current knowledge on *Eumycetozoa* as a reservoir of bioactive compounds, detailing how secondary metabolites—including polysaccharides, amino acids, unsaturated fatty acids, terpenoids, and glycosides—vary across plasmodia, fruiting bodies, and spores. A systematic literature search in major scientific databases accounted for legacy nomenclature and leveraged chemoinformatic tools for compound verification. Our findings reveal 298 distinct metabolites that serve ecological roles in nutrient recycling and interspecies interactions, while also showing promise for controlling agricultural pests and pathogens. Notably, certain glycosides, lectins, and polyketides exhibit antimicrobial or cytotoxic activities, indicating their potential utility in managing these biological challenges. By consolidating current data and emphasizing the wide taxonomic range of *Eumycetozoa*, this review highlights the critical need for comprehensive biochemical and genomic investigations. Such efforts will not only advance our understanding of slime mold metabolomes and their evolutionary significance but also pave the way for innovative, eco-friendly applications.

## 1. Introduction

**Figure 1:**
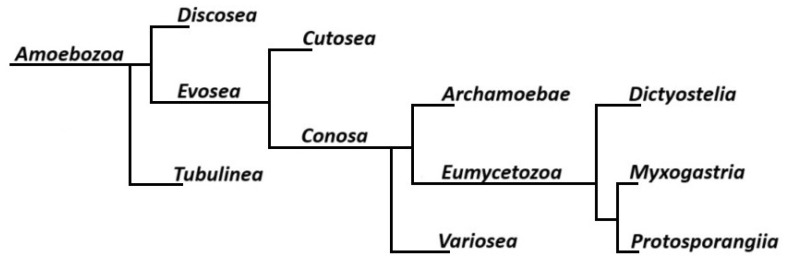
Simplified classification of *Amoebozoa* highlighting the placement of *Eumycetozoa* and its main lineages (*Dictyostelia*, *Myxogastria*, and *Protosporangiida*). Original artwork by Tomasz Pawłowicz, based on the phylogenomic analyses of Tekle et al. (2022) [1], who used 824 single-copy genes (113,910 sites) from 113 taxa, analyzed with the maximum-likelihood (ML) software IQ-TREE (LG + G4 + C60 + F; 1000 ultrafast bootstrap replicates) and RAxML (Randomized Axelerated Maximum Likelihood; PROTGAMMALG4X).

True slime molds (*Eumycetozoa*) are a monophyletic clade within the phylum *Amoebozoa* that encompasses three main lineages: *Myxogastria*, *Dictyostelia*, and *Protosporangiida* (Figure 1) [1]. Although the term “slime molds” has historically referred to a broad spectrum of eukaryotic microorganisms—including those in *Stramenopiles*, *Rhizaria*, *Discoba*, and *Holomycota* [2,3]—current molecular phylogenetics confirms that only *Eumycetozoa* represent the “true slime molds”, unambiguously placed in the supergroup *Amoebozoa* [4]. These organisms are predominantly terrestrial, thriving in habitats rich in decaying organic matter such as forest floors and soils [5]. They play notable ecological roles as decomposers, breaking down leaf litter and wood, and serving as prey for diverse microorganisms [6,7].

Globally, approximately 1100 species of *Eumycetozoa* have been described, underscoring their broad distribution and ecological significance [2,3,8]. Within this clade, *Myxogastria* (often referred to historically as *Myxomycetes*) is perhaps the most widely recognized group, characterized by acellular plasmodial stages and often conspicuous fruiting bodies. Although most slime molds remain microscopic for much of their life cycle, certain members of *Myxogastria* produce visible plasmodial structures and macroscopic fruiting bodies [2]. These structures can arise through the aggregation of uninucleate cells guided by chemical signals (e.g., acrasins) or through cellular fusion, resulting in the large multinucleate plasmodium [8]. Besides their decomposer activity, a subset of slime molds exhibits parasitic behaviors, further highlighting the diversity of their ecological strategies [6].

Historically, slime molds were misclassified as fungi due to the superficial resemblance of their fruiting bodies to fungal sporophores. The earliest documented reference dates to the ninth century, when the Chinese scholar Twang Ching-Shith described a substance resembling *Fuligo septica*, which he dubbed “demon droppings” [9,10]. In Europe, *Eumycetozoa* were first documented in Thomas Panckow’s *Herbarium Portabile* (1654), featuring an illustration likely depicting *Lycogala epidendrum* [10,11]. With the advent of molecular phylogenetics, slime molds were reassigned from the fungal kingdom to the supergroup *Amoebozoa*, supported by genomic data that underscore their distinctive nutritional modes (phagotrophy, pinocytosis, and osmotrophy) [12,13]. This reclassification also aligns with morphological and developmental evidence that sets them apart from fungi despite some shared ecological niches.

Fossil records reveal that *Eumycetozoa* have an ancient lineage marked by remarkable morphological stability. Specimens of genera such as *Stemonitis* and *Arcyria* preserved in Baltic amber date to roughly 50 million years ago, while mid-Cretaceous fossils of *Stemonitis*, estimated to be 100 million years old, exhibit striking morphological stasis [14,15,16]. These findings suggest that survival strategies like cryptobiosis have enabled these organisms to persist across significant geological spans.

**Figure 2:**
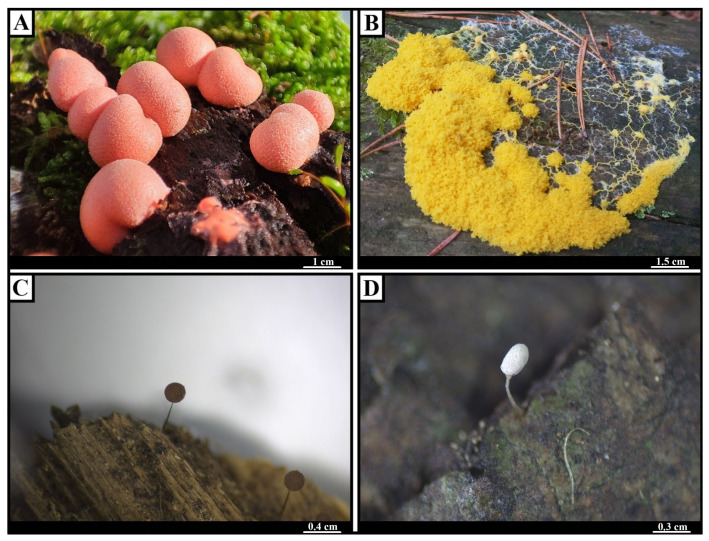
Diverse forms of plasmodia and fruiting bodies observed in various *Eumycetozoa*. (**A**) *Lycogala epidendrum*; (**B**) *Fuligo septica*; (**C**) *Comatricha nigra*; (**D**) *Arcyria cinerea*. Photos by Tomasz Pawłowicz.

Although their evolutionary and ecological importance has long been recognized, the chemical composition of true slime molds remains comparatively underexplored, especially relative to the extensively studied secondary metabolites of fungi [17,18,19,20,21]. *Eumycetozoa* display striking variation in fruiting body size and coloration (Figure 2), ranging from fruiting body structures measuring 50–500 µm (e.g., *Comatricha nigra* (Figure 2C) and *Arcyria cinerea* (Figure 2D)) to larger forms exceeding 1 cm, as seen in *Lycogala epidendrum* (Figure 2A) or *Fuligo septica* (Figure 2B), which can occasionally span over a meter [2,6,10]. Such extreme morphological diversity complicates the collection of sufficient biomass for chemical analyses [22] but also hints at a potentially rich arsenal of bioactive compounds.

**Figure 3:**
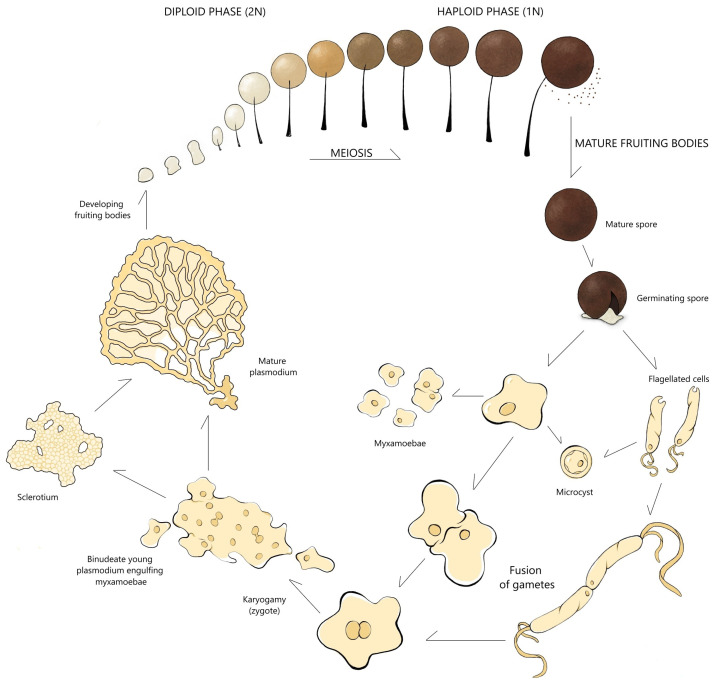
Life cycle of a plasmodial slime mold (*Myxogastria*) based on observations of *Comatricha nigra*. Original work by Igor Żebrowski and Tomasz Pawłowicz, based on Clark et al. [23] and Stephenson et al. [10].

The life cycle of *Myxogastria* exemplifies the complexity found across *Eumycetozoa* typically exhibit a life cycle comprising two trophic stages—uninucleate myxamoebae and multinucleate plasmodia—and a reproductive stage characterized by the formation of spore-bearing structures—fruiting bodies [10]. After germination, spores can differentiate into myxamoebae under moist but not overly saturated conditions or into flagellated swarm cells when exposed to a high humidity environment [10,23]. Under prolonged environmental stress, both the myxamoebae and flagellated cells can transform into microcysts, improving their ability to survive [10,23,24]. Upon encountering favorable conditions, the plasmodium—a dynamic, free-living protoplasmic network—differentiates into fruiting bodies that generate spores. These spores disperse primarily via wind, and when they germinate, new amoeboid cells arise, thus perpetuating the cycle (Figure 3) [10,24,25].

Recent studies have begun to highlight the biochemical and industrial potential of true slime molds. For instance, interactions between *Eumycetozoa* and crop plants or cultivated mushrooms suggest possible agricultural applications [26], while the discovery of novel secondary metabolites (e.g., arcyriarubins, physarigins) emphasizes their promise as sources of pharmacologically active compounds [27,28,29,30]. Despite these advances, significant gaps persist in our understanding of *Eumycetozoa*, including their full taxonomic breadth, biogeography, and chemical diversity. In particular, cryptic or less visible life stages often go undetected in field surveys, potentially overlooking unique metabolite profiles [2,27].

Building on these findings, another emerging yet underinvestigated domain involves the utilization of slime mold-derived bioactive substances in sustainable pest and pathogen control.

Preliminary in vitro assays have indicated that certain extracts from *Eumycetozoa* can inhibit the growth of fungal phytopathogens and other microbial antagonists, suggesting possible roles in integrated pest management regimes [6,31,32,33]. Likewise, some species appear to produce compounds with broad-spectrum activities that could reduce reliance on synthetic agrochemicals [26,27]. Despite these promising insights, comprehensive field trials and toxicity assessments remain scarce, and the underlying mechanisms of action are often poorly understood.

Accordingly, this review aims to synthesize existing knowledge of *Eumycetozoa* as producers of bioactive compounds, with a particular emphasis on the various life-cycle stages in which these substances appear. By combining the historical background, ecological context, and the latest findings on chemical constituents, we seek to illuminate the biochemical potential of true slime molds, identify current research gaps, and propose avenues for further studies in both fundamental and applied contexts. In doing so, particular attention is given to their potential role in sustainable pest and pathogen management, where *Eumycetozoa*-derived compounds may offer eco-friendly alternatives to conventional control strategies.

## 2. Methods of Literature Review

This review synthesizes available information on *Eumycetozoa*—the monophyletic clade within the phylum *Amoebozoa* that includes *Myxogastria*, *Dictyostelia*, and *Protosporangiida*. Given the historical and widespread usage of terms such as “*Myxomycetes*” and “slime molds” in the literature, our search strategy encompassed both the formal taxonomic name (*Eumycetozoa*) and these common synonyms to ensure comprehensive coverage of relevant publications. In addition, special emphasis was placed on identifying reports or preliminary findings concerning slime mold-derived compounds in pest and pathogen control, thereby allowing the subsequent discussion to address this emerging application.

### 2.1. Literature Search and Data Collection

A comprehensive literature search was conducted using multiple major scientific databases, including Web of Science [34], Scopus [35], PubMed [36], ScienceDirect [37], and SpringerLink [38]. To ensure broader coverage of potentially overlooked materials, additional searches were performed in multidisciplinary repositories such as Google Scholar [39].

Keywords and phrases were selected to capture both the contemporary taxonomic framework of true slime molds (*Eumycetozoa*) and older classifications that previously placed these organisms in other kingdoms (particularly fungi). The literature search employed single and compound names, with particular emphasis on two overarching thematic categories:

- Organisms: “*Eumycetozoa*”, “*Myxogastria*”, “*Myxomycetes*”, “slime molds”, “*Dictyostelia*”, “*Protosporangiida*”, and equivalent descriptors used in older studies.
- Chemical substances: “bioactive compounds”, “secondary metabolites”, “biologically active substances”, “natural products”, and related terms found in both contemporary and historical nomenclatures.

Representative queries combined these keywords to ensure comprehensive retrieval of relevant publications (e.g., “*Myxomycetes* bioactive compounds”, “slime mold secondary metabolites”, “*Eumycetozoa* chemical diversity”, “slime mold pest control”, “*Myxomycetes* pathogen inhibition”, or “*Eumycetozoa* agricultural applications”).

To refine our search specifically for bioactive compounds, we performed an initial screening of titles and abstracts, selecting works that directly discussed the chemical composition of slime molds. Through this process, 95 references were ultimately identified that focus on *Eumycetozoa* metabolites. The remaining cited materials provide essential context regarding slime mold biology, ecology, life cycles, and biodiversity, as well as references to the scientific databases consulted.

Given the recent interest in environmentally sound agrochemicals, we also reviewed studies that mentioned or tested slime mold metabolites for their antimicrobial or pesticidal properties. This inclusive approach was designed to capture both recent publications and legacy literature, acknowledging that earlier work may reference slime molds under superseded classifications. Consequently, the final dataset spans a wide chronological range, permitting a thorough assessment of the chemical diversity documented within *Eumycetozoa*.

### 2.2. Compound Identification and Verification

Information on nomenclature, molecular structures, and formulas of identified compounds was collated using chemoinformatics resources, with the National Center for Biotechnology Information (NCBI) PubChem database [40] serving as the principal reference. Supplemental databases such as Chemical Entities of Biological Interest (ChEBI) [41], the National Institute of Standards and Technology (NIST) database [42], the Chemical Abstracts Service (CAS) registry [43], and The Pherobase [44] were consulted if a specific compound record was unavailable in PubChem.

Several unique metabolites originally reported as novel to science—such as arcyriacyanin A and physarochrome A—remain absent from major chemical repositories due to their recent discovery. For these compounds, we retained the nomenclature utilized in the original publications to ensure consistency with existing literature.

### 2.3. Taxonomic Nomenclature

Slime mold taxonomy follows the International Code of Nomenclature for Algae, Fungi, and Plants (ICN) [45]. Synonym verification and the most recent taxonomic updates were confirmed using multiple authoritative databases, including Index Fungorum [46], the USDA Fungal Databases [47], and MYCOBANK [48]. Throughout this review, “*Eumycetozoa*” is used to collectively refer to *Myxogastria*, *Dictyostelia*, and *Protosporangiida*.

### 2.4. Developmental Stages and Metabolites Surveyed

Because *Eumycetozoa* exhibit complex life cycles, data on bioactive compounds were gathered from all known developmental stages, including the plasmodium, fruiting body, slime tracks, cellular stage (myxamoebae), spores, and sclerotium. By integrating information across these stages, we aim to present a comprehensive overview of the chemical diversity within true slime molds. This approach also allowed us to note any developmental-phase specificity of compounds that have been explored—or could potentially be explored—for pest or pathogen inhibition.

### 2.5. Classification of Identified Compounds

In accordance with internationally recognized chemical nomenclature standards (e.g., IUPAC) and to ensure a robust, biosynthetically and structurally coherent framework, all retrieved chemical entities were reorganized into eight principal categories. This updated classification embraces both discrete, well-characterized metabolites (e.g., specific amino acids and identified fatty acids) and broader classes of structurally or functionally related molecules (e.g., diverse quinone pigments and unique *Eumycetozoa*-specific secondary metabolites). By adhering to these international guidelines, we aimed to harmonize previously fragmented groupings and to establish clear, consistent criteria for compound categorization:

(a) **Carbohydrates and Their Derivatives**
  Encompasses monosaccharides, oligosaccharides, polysaccharides, glycosylated metabolites, and various sugar acids critical for structural and metabolic roles in *Eumycetozoa*.
(b) **Amino Acids, Peptides, and Proteins**
  Includes both proteinogenic and non-proteinogenic amino acids, low-molecular-weight peptides, and large protein complexes such as enzymes or structural proteins.
(c) **Lipids**
  Groups all relevant lipid classes, from saturated and unsaturated fatty acids to glycerophospholipids, sterols, steroids, and terpenoids, reflecting their importance in membrane architecture, signaling, and energy storage.
(d) **Polyphenols, Quinones, and Related Polyketides**
  Covers aromatic metabolites often derived from polyketide synthase pathways, featuring phenolic and quinone functionalities. Such compounds frequently exhibit notable bioactivities (e.g., antioxidant, antimicrobial, and cytotoxic).
(e) **Pigments**
  Focuses on colored compounds, including carotenoids, melanin-related pigments, and other chromophores (e.g., arcyriarubins and fuligorubins) implicated in protective functions or developmental processes.
(f) **Alkaloids and Indole Derivatives**
  Comprises nitrogen-containing secondary metabolites, typically bearing heterocyclic scaffolds (e.g., staurosporines and arcyriaflavins), many of which show potent biological effects, such as antimicrobial or cytotoxic activities.
(g) **Polyenes and Polyacetylenes**
  Encompasses molecules characterized by multiple conjugated double bonds or acetylenic linkages, often displaying pronounced bioactivity and distinct spectroscopic properties.
(h) **Other** ***Eumycetozoa*****-Specific Secondary Metabolites**
  Serves as a repository for specialized or structurally unique compounds exclusive to certain *Eumycetozoa* lineages (e.g., acyltetramic acids, heterocyclic antibiotics, and discoidin-type proteins), which do not conform neatly to the preceding categories.

This approach consolidates and clarifies compound assignments, ensuring that molecules with shared biosynthetic pathways or core structural motifs are classified together. Moreover, provisional or partially characterized substances are accommodated within an appropriate broad grouping, pending further elucidation of their precise structures and biological functions. Coupled with targeted literature searches for agroecological applications, this classification also helps pinpoint which compound families may hold promise in integrated pest management or pathogen control strategies, a focus that will be revisited in the Discussion. As a result, this methodology aligns with the best practices in chemical taxonomy and facilitates a more accurate interpretation of the metabolic diversity found in *Eumycetozoa*.

## 3. Literature Review Results

The results presented here include comprehensive compilations and detailed descriptions of chemical compounds identified in *Eumycetozoa* at different developmental stages. Based on a comprehensive review of the existing literature, the compounds were systematically categorized to reflect their structural and biosynthetic properties. This classification framework is organized into eight distinct chemical categories that allow for an in-depth investigation of metabolomic diversity in true slime molds. Each category describes a broad class of compounds that are further subdivided as necessary to reflect specific structural or functional features. The following subsections provide a thorough examination of each chemical class and highlight the distribution and potential bioactivities of the identified compounds in different life cycle stages of *Eumycetozoa*.

### 3.1. Carbohydrates, Their Derivatives, and Glycoconjugates

Carbohydrates, their derivatives, and glycoconjugates constitute a critical group of biomolecules involved in numerous structural and metabolic processes across the life cycles of true slime molds (*Eumycetozoa*). Although investigations dedicated solely to carbohydrate biosynthesis remain relatively limited, existing data reveal that these organisms produce both mono- and polysaccharides in varying proportions, depending on developmental stage and environmental conditions. Many of these compounds occur as extracellular slime constituents or form part of complex protein–carbohydrate assemblies in the plasmodium and fruiting body.

Early work by Simon and Henney [49] demonstrated that extracellular slime in *Physarum flavicomun*, *Ph. polycephalum*, and *Ph. rigidum* consisted largely of galactose-containing glycoproteins. Depending on the growth medium, additional neutral sugars such as glucose or mannose appeared to modulate the composition of the slime matrix. Subsequent research highlighted that slime track exopolysaccharides (EPSs) in both *Ph. polycephalum* and *Physarella oblonga* can contain rhamnose, galactose, and glucose, with rhamnose being predominant in *Phy. oblonga* [50]. In *Dictyostelium discoideum*, meanwhile, Yamada et al. [51] revealed a broader range of extracellular polysaccharides, identifying glucose, galactose, mannose, glucuronic acid, and galacturonic acid, as well as glucosamine residues. Further emphasizing the diversity of carbohydrate metabolism in *D. discoideum*, Ceccarini and Filosa [52] described distinct developmental fluctuations in trehalose content, which rises sharply in the transition from vegetative growth to spore formation.

Beyond these extracellular matrices, slime molds can synthesize specialized polysaccharides with unique structural features or biological functions. Farr and Horisberger [53], for instance, isolated a sulfated β-*D*-galactan from the nuclei of *Ph. polycephalum*, distinguished from extracellular galactan by a higher sulfate content and occasional (1 → 3) and (1 → 6) linkages. In addition, Murakami-Murofushi et al. [54] reported that plasmodial protein complexes in *Ph. polycephalum* harbor mannose, glucosamine, fucose, and glucose, highlighting the importance of glycosylation in intracellular enzymes. Coupled with these findings, the work of Řezanka and Dvořáková [55] introduced the concept of polypropionate lactone glycosides—namely lycogalinosides A and B—in *Lycogala epidendrum*, compounds carrying 2-deoxy-fucopyranosyl or gulopyranosyl moieties. Their structural uniqueness and inhibitory activity against Gram-positive bacteria subsequently garnered attention in review articles by Dembitsky et al. [56], as well as in broader physiological studies [57,58], which affirmed the occurrence of these glycosides in related species such as *Dictyostelium rhizoposium*.

Taken together, these data underscore the compositional complexity of carbohydrates in true slime molds, where monosaccharides, polysaccharides, and glycosides contribute to both extracellular matrices and intracellular structural elements. Moreover, shifts in carbohydrate profiles at different points in the life cycle support the hypothesis that these compounds may serve roles not only in nutritional storage but also in defense, cellular signaling, and morphogenesis. Table 1 outlines the main carbohydrates and related glycoconjugates identified to date, documenting the respective species, developmental stages, and references detailing their discovery.

### 3.2. Amino Acids, Peptides, and Proteins

Amino acids, peptides, and macromolecular proteins in *Eumycetozoa* are categorized into proteinogenic and non-proteinogenic amino acids, as well as enzymes and other macromolecular proteins. The subsequent subsections provide detailed classifications and descriptions of these groups.

#### 3.2.1. Proteinogenic and Non-Proteinogenic Amino Acids

Proteinogenic amino acids are those commonly found in proteins. Non-proteinogenic amino acids can be post-translationally modified residues or entirely distinct structures with specialized functions (e.g., secondary metabolites and intermediates in metabolic pathways).

Amino acids and their polymeric forms (peptides and proteins) are central to nearly all physiological processes in true slime molds (*Eumycetozoa*). They participate in fundamental metabolic pathways, form structural filaments such as actomyosin, and serve as precursors or regulatory molecules in various developmental transitions. Investigations of amino acid composition in both plasmodial and cellular slime molds have revealed dynamic changes linked to spore formation, germination, and cyst development, implying that free amino acid pools may play active roles in energy metabolism and morphogenesis.

In one series of studies, Ennis [59] monitored free amino acid concentrations during spore germination in *Dictyostelium discoideum* and reported marked fluctuations in alanine, aspartic acid, threonine, and several other amino acids as dormant spores transitioned to amebae. Using synchronous germination conditions in phosphate buffer, spores were collected at defined developmental stages, lysed in trichloroacetic acid, and analyzed through amino acid chromatography. Notably, proline exhibited a unique pattern, being present in relatively low amounts in dormant spores but becoming more abundant during early germination in *D. discoideum*. Parallel analyses of cyst germination in *Polysphondylium pallidum* confirmed similar shifts in amino acid pools, reinforcing the hypothesis that internal reserves of specific amino acids provide both carbon skeletons and nitrogen sources during the initial phases of outgrowth.

Threlfall’s earlier work [60] focused on *Physarum polycephalum*, assessing free amino acid profiles in microplasmodia and microcysts throughout the mitotic cycle. By cultivating plasmodia in controlled media and sampling at discrete cell cycle stages, it was demonstrated that aspartic acid, glutamic acid, lysine, and leucine typically comprised the largest fraction of the total amino acid pool, whereas proline followed a distinct trajectory during S-phase and telophase. These findings highlighted the temporal regulation of amino acid metabolism in *P. polycephalum*, emphasizing that fluctuations in pools of essential and non-essential amino acids reflect the biosynthetic and energetic demands of rapidly dividing or differentiating cells.

Collectively, these data indicate that slime molds possess finely tuned mechanisms for modulating free amino acids across different life-cycle stages. Table 2 summarizes the principal proteinogenic amino acids detected in *D. discoideum* and *P. polycephalum* spores and microcysts, along with the studies in which they were identified. This overview underscores the integral role of amino acids in slime mold development and suggests that future research may uncover additional non-proteinogenic variants and specialized peptide-based signaling pathways.

#### 3.2.2. Enzymes, Peptides, and Macromolecular Proteins

Enzymes, peptides, and other macromolecular proteins serve as catalysts, structural scaffolds, or mediators of cell adhesion in true slime molds (*Eumycetozoa*). These high-molecular-weight biomolecules participate in diverse processes related to cellular metabolism, morphogenesis, and environmental adaptation. Examples include key metabolic enzymes (e.g., aminotransferases and dehydrogenases) and specialized proteins like myosin, actin, or lectins that facilitate motility, cytokinesis, or intercellular recognition.

Among the earliest investigations, Firtel and Brackenbury [61] conducted a partial characterization of several amino-acid-metabolizing enzymes in *Dictyostelium discoideum*, using spectrophotometric assays to monitor alanine aminotransferase (ALT), aspartate aminotransferase (AST), and aminopeptidase activities in crude extracts. These activities were shown to increase during development under starvation conditions, indicating a crucial role for protein turnover in differentiation. In parallel, Spudich [62] and Clarke [63] applied biochemical fractionation, sucrose extraction, and electron microscopy to isolate actomyosin components from *D. discoideum* plasmodia. They demonstrated that myosin II and actin form bipolar filaments and display ATPase activity akin to muscle myosin, a finding further elaborated by Yumura [64]. Studies of lactate dehydrogenase and glutamate dehydrogenase in *D. discoideum* also revealed distinct regulatory profiles, with some enzymes remaining largely unaffected by developmental cues [61]. More recently, the presence of delta-5-fatty acid desaturase activity was reported in *D. discoideum*, inferred from analyses of polyunsaturated fatty acid (PUFA) biosynthesis, and underscored by both experimental findings [65] and review-based confirmations [66].

Investigations in *Physarum polycephalum* have uncovered unique macromolecular proteins as well. Yokota et al. [67] purified pallidin and a hemagglutinin named physarumin, each presumably involved in cell–cell or cell–substrate interactions. The same study also identified discoidin in *D. discoideum*, a lectin-like molecule implicated in adhesion.

In addition, Shimomura et al. [68] explicitly identified a non-ribosomal peptide (NRP) known as glorin in *Polysphondylium violaceum*. Through spectrometric analysis and chemotaxis assays, its structure was elucidated and its role established as the acrasin, i.e., the primary signaling molecule directing aggregation in this organism. Although NRPs are well-characterized in other organisms (e.g., fungi), no additional NRPs have been conclusively identified in slime molds beyond glorin. This gap underscores an underexplored dimension of slime mold secondary metabolism, where future studies may reveal further peptide-based signaling mechanisms or bioactive compounds.

As summarized in Table 3, these diverse proteins, enzymes, and peptides highlight the metabolic versatility of slime molds across different life-cycle stages, from plasmodial growth and fruiting-body formation to spore maturation.

### 3.3. Lipids

Lipids in *Eumycetozoa* comprise hydrophobic and amphipathic molecules essential for energy storage, membrane biogenesis, and cellular signaling. The primary lipid classes identified include saturated and unsaturated fatty acids, glycerophospholipids, sterols, and steroids, as well as terpenoids.

#### 3.3.1. Saturated Fatty Acids

Saturated and branched-chain fatty acids (BCFAs) are essential lipid components that serve as metabolic fuels, structural membrane constituents, and potential chemotaxonomic markers. In *Eumycetozoa*, these fatty acids vary from short-chain to very-long-chain derivatives and frequently exhibit iso- or anteiso-branching patterns. Early work by Davidoff and Korn [69] in *Dictyostelium discoideum* used gas–liquid chromatography (GLC) to identify minor proportions of several saturated acids (notably heptadecanoic and nonadecanoic), highlighting the occurrence of uncommon odd-chain homologs. Subsequent studies by Rézanka [70] and Dembitsky et al. [56] broadened this scope, documenting up to 21 distinct saturated and branched-chain fatty acids across multiple slime mold genera, including *Arcyria*, *Fuligo*, *Lycogala*, *Physarum*, and *Trichia*. Their analyses primarily employed capillary gas chromatography—mass spectrometry (GC–MS) of fatty acid derivatives, revealing that fruiting bodies often contain a range of iso- and anteiso-branched compounds. Complementary work by Comes and Kleinig [71] demonstrated iso-hexadecanoic acid in *Physarum polycephalum* plasmodia, further confirming that branched-chain variants are not restricted to fruiting stages.

Davidoff and Korn [69,72] also found common straight-chain acids (lauric, myristic, palmitic, and stearic) in *D. discoideum*, while later assays by Long and Coe uncovered similar saturated profiles but in lower proportions compared to unsaturated or cyclopropane derivatives. In *P. polycephalum*, iso-tetradecanoic, anteiso-pentadecanoic, and iso-hexadecanoic acids were identified via GC–MS-based phospholipid fractionation [71], strengthening evidence that multiple branched-chain structures are biosynthesized or selectively incorporated in true slime molds. The comprehensive survey by Dembitsky et al. [56] further validated these findings and integrated earlier literature, affirming that branched-chain acids such as 10-methyldodecanoic and 14-methylpentadecanoic occur consistently among fruiting bodies of species like *Arcyria cinerea* and *Trichia favogiena*.

As summarized in Table 4, these saturated and branched-chain fatty acids can be found in both plasmodia and fruiting bodies of diverse *Eumycetozoa*. Their presence not only underlines the biochemical adaptability of slime molds but also highlights potential evolutionary links to other protozoan taxa with similar lipid profiles.

#### 3.3.2. Unsaturated Fatty Acids

Unsaturated fatty acids (UFAs) represent a broad class of lipids that incorporate one or more carbon–carbon double bonds into their hydrocarbon chain. In slime molds (*Eumycetozoa*), these compounds function as integral membrane constituents, precursors in signaling cascades, and potential chemotaxonomic markers. Many species produce both common mono- and polyunsaturated fatty acids as well as unusual dienoic or trienoic acids with non-methylene-interrupted double bonds (NMID FAs). Such diversity underscores a complex interplay of desaturase enzymes, environmental influences, and potential bacterial contributions to slime mold lipidomes.

Early investigations into unsaturated fatty acids in cellular slime molds began with Davidoff and Korn [69], who used GLC to examine *Dictyostelium discoideum* and reported a surprising abundance of monoenes (e.g., palmitoleic acid, 9–16:1) and unusual dienes, such as 5,9–16:2. These findings were expanded in subsequent work [69] that detailed minor but structurally intriguing acids like 5,9-heptadecadienoic acid and 5,11-octadecadienoic acid. Korn and co-workers later assessed the lipid composition of plasmodial slime molds, revealing that *Physarum polycephalum* differed from *D. discoideum* by synthesizing robust levels of linoleic acid (9,12–18:2) and oleic acid (9–18:1), yet lacking certain NMID variants observed in cellular forms [72]. Around the same time, Long and Coe [73] explored how *D. discoideum* transitions to mature sorocarps, noting increased proportions of octadeca-5,11-dienoic acid in the later developmental stages and attributing certain cyclopropane or branched unsaturated lipids to bacterial ingestion.

Systematic studies by Rézanka [70], employing GC–MS of fatty acid oxazoline derivatives, provided a comprehensive survey of unsaturated fatty acids in nine different myxomycete genera (e.g., *Arcyria*, *Fuligo*, *Lycogala*, *Physarum*, *Trichia*, and *Lindbladia*). This work cataloged a wide range of monoenes (e.g., 7–20:1, 11–18:1, 13–22:1), conjugated or non-methylene-interrupted dienes (5,9–16:2, 5,11–20:2), and high-degree polyenes such as arachidonic acid (5,8,11,14–20:4) and docosahexaenoic acid (22:6). In parallel, additional investigations demonstrated that these unsaturated profiles often shift according to life-cycle stage—whether plasmodia, fruiting bodies, or amoebal forms—and can be influenced by nutritional parameters (for example, ingestion of certain bacterial strains). Dembitsky [56] consolidated these earlier findings into a broad review of secondary metabolites in slime molds, underlining that high levels of alpha-linolenic acid (9,12,15–18:3), Mead acid (5,8,11–20:3), and other polyunsaturated structures consistently appear in fruiting bodies across multiple taxa.

More specialized efforts have refined this knowledge base. Saito and Ochiai [65] studied cyclopropane FAs and a $\Delta^{5}$-fatty acid desaturase in *Polysphondylium pallidum*, thereby illuminating the mechanisms by which slime molds introduce specific double bonds during aggregation. Misono et al. [74] characterized docosadienoic (C_22_:2) and docosatetraenoic (C_22_:4) acids in *Lindbladia tubulina*, confirming their presence through GC–MS-based structural assignments. Studies on *Trichia favogiena* and *T. varia* showed that 5,9–18:2, 5,11–18:2, and 5,11,14–20:3 represent further examples of non-methylene-interrupted PUFAs unique to certain slime molds [56,70].

Collectively, these investigations emphasize the biochemical versatility of *Eumycetozoa*, which produce an array of unsaturated fatty acids spanning common monoenes (e.g., oleic, vaccenic) to highly unsaturated moieties such as eicosapentaenoic (EPA) and docosahexaenoic (DHA). As detailed in Table 5, many of these compounds arise in the fruiting-body stage, although a few (like palmitoleic and vaccenic acids) also occur in plasmodia or vegetative amoebae. The abundance of unusual double-bond positions, particularly $\Delta^{5,9}$ or $\Delta^{5,11}$, may serve as taxonomic indicators and reveal distinctive enzymatic pathways. Ongoing research continues to explore how culture conditions, bacterial prey, and genetic variation influence these lipid profiles, thereby expanding our understanding of slime mold lipid metabolism and its broader biological significance.

#### 3.3.3. Glycerophospholipids and Related Phospholipids

Glycerophospholipids represent the major polar lipids in eukaryotic membranes, distinguished by their glycerol backbone, attached fatty acid chains, and diverse phosphorylated head groups. In true slime molds (*Eumycetozoa*), these compounds occur in diacyl, alkyl-acyl, and plasmalogen variants, often displaying considerable structural complexity linked to specific developmental stages or environmental conditions.

One of the earliest detailed investigations of slime mold phospholipids was carried out by Davidoff and Korn [69], who examined *Dictyostelium discoideum* (including an aggregateless mutant) through lipid extractions followed by thin-layer chromatography (TLC) and GLC. They identified phosphatidylethanolamine (PE), phosphatidylcholine (PC), and lysophosphatidylethanolamine (LPE) among the principal lipid fractions, noting their distribution in various subcellular fractions. In a separate line of research, Comes and Kleinig [71] characterized the phospholipid composition of *Physarum polycephalum* plasmodia using two-dimensional TLC. Their study revealed phosphatidylcholine, phosphatidylethanolamine, and phosphatidylinositol as the predominant classes, with smaller proportions of phosphatidic acid and cardiolipin also detected. Notably, phosphatidylserine was absent from the *P. polycephalum* extracts they analyzed. Subsequent work indicated that phosphatidic acid occurs in additional taxa, including certain *Tubifera* and *Physarella* species, with later efforts providing direct evidence for its role in phospholipase-mediated remodeling [58,76].

Cardiolipin and phosphatidylserine, although occasionally minor constituents, have been reported in several genera of *Myxogastria*. In earlier publications by Steglich and co-workers [77,78], cardolipins (and occasional phosphatidylserine) were found in *Arcyria* and related taxa, often in low abundance but nonetheless indicative of specialized mitochondrial lipid pools. Likewise, phosphatidylinositol has been documented through isolation and chemical characterization in *Fuligo septica*, *Physarum* species, and *Trichia varia* [75,79], affirming its conserved presence in both plasmodial and fruiting-body stages.

In *Dictyostelium discoideum*, plasmalogen-type glycerophospholipids have been described by Kikuchi et al. [80], who applied extraction and chromatographic fractionation to detect 1-O-alk-1’-enyl-2-acyl-sn-glycero-3-phosphoethanolamine (plasmenylethanolamine), whereas 2-acyl-1-alkyl-sn-glycero-3-phosphocholine was found in certain culture conditions. Plasmalogen phospholipids likewise appear in *Arcyria denudata* and *Lycogala flavoscum*, as inferred from structural analyses of alk-1-enyl moieties. In addition, Nowak and Steffan [81] isolated polycephalin B and C from illuminated plasmodia of *Physarum polycephalum*, suggesting a potential role for these atypical tetramic acid-linked phospholipids in blue-light responses. Subsequent authors confirmed the presence of these polycephalins in *Fuligo septica* and other slime molds [58,82], and a recent review on their biotechnological potential was provided by Stoyneva-Gärtner et al. [66].

A broader overview of glycerophospholipid classes across multiple slime mold taxa, including references to alkyl-acyl forms and lysophospholipids, can be found in the comprehensive compilation by Dembitsky et al. [56]. That review collates original observations from the aforementioned studies and additional sources, underscoring how diacyl glycerophosphoethanolamine, plasmalogen phosphatidylcholine, and cardiolipin commonly recur in diverse genera (e.g., *Arcyria*, *Fuligo*, *Physarum*, *Trichia*). Table 6 summarizes the main glycerophospholipid types reported to date, highlighting the methodological breadth—from GLC of fatty acid methyl esters to 2D-TLC of intact phospholipids—employed to elucidate their distribution and structural features in *Eumycetozoa*.

#### 3.3.4. Sterols, Steroids, and Terpenoids

Sterols, steroids, and terpenoids represent a diverse range of isoprenoid compounds that fulfill multiple biological roles in eukaryotes, including membrane stabilization, hormone signaling, and defense responses. In true slime molds (*Eumycetozoa*), early work focused on canonical sterols such as stigmasterol and β-sitosterol, but more recent investigations have uncovered specialized terpenoids (e.g., rearranged triterpenes, sesquiterpenes, and glycosidic lactones) that exhibit distinctive structural motifs and potential bioactivities.

Much of our current understanding of slime mold sterols stems from original quantitative and qualitative surveys carried out on *Physarum* species. Bullock and Dawson [84] analyzed plasmodial cultures of *Physarum polycephalum* and *Physarum flavicomun* GLC and nuclear magnetic resonance (^1^H/^13^C NMR), identifying poriferasterol, 22-dihydroporiferasterol, and lanosterol as the primary components. In a parallel study, Lenfant et al. [85] examined axenic *P. polycephalum* through alkaline hydrolysis and alumina column chromatography, confirming the presence of stigmasterol, β-sitosterol, stigmastanol, campesterol, campestanol, and cholesterol by mass spectrometry (MS) and chiral circular dichroism. Additional sterols, including 24-methylene-24-dihydrolanosterol, were subsequently documented by Comes and Kleinig [71] in *P. polycephalum* plasmodia, underscoring the intricate blend of “plant-like” (e.g., sitosterol) and “animal-like” (e.g., lanosterol) sterols in this taxon.

More nuanced comparisons of sterol composition in the haploid myxoamoebae and diploid plasmodia of *P. polycephalum* were provided by Murakami-Murofushi et al. [86]. Employing GC-MS, and ^1^H/^13^C NMR, they found poriferasterol, δ15-ergostenol, and 22-dihydroporiferasterol at differing ratios between life-cycle stages, suggesting a regulatory role for these sterols in membrane functions or developmental transitions. Ishibashi [82] later confirmed the occurrence of poriferasterol and ergostenol in *Physarum* plasmodia in a broader survey of myxomycete metabolites, while Dembitsky [56] and Stoyneva-Gärtner et al. [66] cited many of these same sterols in their reviews, integrating data from earlier original studies. Investigations beyond *Physarum* have revealed that certain *Didymium* species, such as *D. minus*, produce sterols like brassicasterol, clionasterol, and poliferasterol in their fruiting bodies [82], indicating a sterol repertoire overlapping with both “plant-type” and “protozoal-type” pathways. For terpenoids, Sasaki et al. [87] isolated mucoroidiol (a protoilludane sesquiterpene) and firmibasiol (a geranylated bicyclogermacranol) from *Dictyostelium mucoroides* and *Dictyostelium firmibasis*, respectively, using methanol extractions followed by silica- and ODS-column chromatography. Kamata et al. [88] discovered tubiferal A and tubiferal B in field-collected *Tubifera dimorphotheca*, employing acetone/methanol extraction and high-performance liquid chromatography (HPLC) purification. These rearranged triterpenoids exhibit unique 9,10-secocycloartane frameworks and, as later shown by Ishibashi [79], can display cytotoxic or multidrug-resistance-reversing properties.

Further expanding the known terpenoid inventory, Řezanka [89] reported lycoperdinosides A and B as six-membered lactone glycosides from *Tubifera* fruiting bodies (though initially associated with *Enteridium lycoperdon*). Detailed spectroscopic work, such as ^1^H/^13^C NMR, MS, infrared spectroscopy (IR), and ultraviolet spectroscopy (UV), indicated unusual glycosidic linkages that have not been widely observed in other eukaryotic microbes. Subsequent compilations by Li et al. [58] and Stoyneva-Gärtner et al. [66] reinforced the breadth of slime mold terpenoid chemistry, citing these findings alongside established sterols like β-sitosterol, campestanol, stigmasterol, and lanosterol [56,85].

Taken together, these original studies and later reviews demonstrate that slime molds produce both widely recognized sterols (e.g., cholesterol, β-sitosterol) and distinctive terpenoid scaffolds (e.g., tubiferal-type triterpenoids and geranylated bicyclogermacranols). Table 7 collates the principal steroids and terpenoids identified thus far, referencing the key methodological approaches—ranging from GLC to ^1^H/^13^C NMR for sterol profiling to specialized HPLC isolation for rearranged triterpenes—that have illuminated the complex isoprenoid pathways within *Eumycetozoa*.

### 3.4. Polyphenols, Quinones, and Related Polyketides

The category of aromatic compounds within *Eumycetozoa* has been subdivided into three distinct groups: polyphenols, quinones, and other polyketides. Polyphenols encompass compounds derived from the acetate or shikimate pathways, characterized by multiple phenolic hydroxyl groups. Quinones are defined by their quinone structures, which are pivotal for various redox reactions. The other polyketides category includes a diverse array of secondary metabolites synthesized via polyketide synthase enzymes, exhibiting a wide range of biological activities.

#### 3.4.1. Polyphenols

Polyphenols are a structurally diverse class of secondary metabolites characterized by multiple phenolic groups. In true slime molds (*Eumycetozoa*), these compounds may influence developmental processes such as stalk and spore differentiation, while also exhibiting potential antimicrobial or cytotoxic activities. Recent investigations have revealed that certain *Dictyostelium* species synthesize polyphenols structurally related to polyketide-derived factors, whereas others produce biphenyl or terphenyl derivatives with possible bioactive functions.

The best-studied example is 4-methyl-5-pentylbenzene-1,3-diol (MPBD), originally identified in *Dictyostelium discoideum* by Saito et al. [90], who employed solvent extraction of culture media and subsequent chromatographic isolation to uncover new differentiation factors in mutant strains deficient in DIF-1 production. Kubohara and Kikuchi later discussed MPBD in a broader review of *Dictyostelium* metabolites [91], emphasizing its roles in stalk cell formation and additional developmental pathways. Synthetic approaches to MPBD, including structure–activity relationship studies, were subsequently reported by Murata et al. [92] to confirm its antimicrobial and differentiating effects.

In a separate line of research on *Dictyostelium polycephalum*, Kikuchi et al. [93] extracted fruiting bodies with methanol, partitioned the extracts, and purified them via silica-gel and octadecyl (ODS) column chromatography. This work led to the isolation of dictyobiphenyl A, dictyobiphenyl B, and two *m*-terphenyl analogues (dictyoterphenyl A and dictyoterphenyl B). These aromatic polyphenols were structurally characterized by ^1^H/^13^C NMR spectroscopy and high-resolution MS, and their selective antiproliferative activities in cultured tumor cells were evaluated. An overview of these and other polyphenols, along with their species of origin and the corresponding references, is provided in Table 8.

Polyphenols comprise multiple phenolic rings and can include flavonoid-like structures, phenolic acids, and other derivatives. These compounds often exhibit strong antioxidant activity and can participate in defense responses or signaling processes.

#### 3.4.2. Quinones

Quinones and related polyketides encompass a wide variety of structurally diverse metabolites synthesized via the polyketide pathway. In true slime molds (*Eumycetozoa*), many of these compounds appear as colored pigments, often with pronounced antimicrobial or cytotoxic activities. They can be naphthoquinones (e.g., cribrariones), anthraquinone-like scaffolds, or more complex frameworks (e.g., bisindoles bearing quinone moieties). Some originate from field-collected fruiting bodies, while others have been isolated from cultured plasmodia. Early work by Steglich and co-workers [78] demonstrated the presence of 2,3,5-trihydroxynaphthoquinone in *Trichia* fruiting bodies, whereas subsequent surveys by Ishibashi et al. [82] confirmed additional pigments (e.g., homotrichione) in *Metatrichia* and *Didymium*. Later reviews by Dembitsky [56] and Stoyneva-Gärtner et al. [66] compiled many of these findings, integrating them with newer reports. Steffan [94] likewise investigated *Physarum polycephalum* plasmodia, identifying physarorubinic acids A/B and physarochrome A. More detailed structural and biosynthetic insights came from Eisenbarth and Steffan [95], who characterized chrysophysarin A in *P. polycephalum*, verifying its polyketide origin via isotopically labeled precursors.

Quinones and derivatives also feature prominently in *Lycogala epidendrum*, where studies by Fröde et al. [96] and Kamata et al. [97] revealed the lycogalic acids (including their dimethyl esters) as key polyketide constituents. Buchanan et al. [98] further explored these dimethyl ester variants using HPLC and spectroscopic analysis. In parallel, Hosoya et al. [30] and Hashimoto et al. [29] focused on bisindole-type metabolites like lycogarubin A/B/C, demonstrating their potent cytotoxic or kinase-inhibitory properties. Additional modifications of lycogarubins (e.g., certain analogues) were unveiled through the spectroscopic work of Ishibashi [79,82] and again referenced in the comprehensive overviews by Stoyneva-Gärtner et al. [66].

Beyond *Lycogala*, naphthoquinone pigments such as lindbladione and dihydrolindbladione have been documented in *Lindbladia tubulina*, often correlating with antibacterial potential [74,79]. In *Cribraria* species, cribrarione A/B/C emerged as antimicrobial dihydrofuranonaphthoquinones [82], while *Trichia* and *Metatrichia* taxa furnished homotrichione, trichione, and vesparione—again highlighting the broad structural range of polyketide-derived quinones in *Myxogastria* [66,78]. Table 9 compiles these notable quinones and polyketide analogues, referencing both the original isolation studies and secondary reviews.

#### 3.4.3. Other Polyketides

Other polyketides represent a heterogeneous category of specialized metabolites derived primarily from polyketide synthase pathways. These structures can feature macrocyclic lactones, difuran derivatives, or various extended aliphatic side-chains, often exhibiting potent bioactivities such as antimicrobial or cytotoxic effects. In true slime molds (*Eumycetozoa*), multiple research groups have employed chromatographic isolation (e.g., silica, ODS, and Sephadex LH-20) and advanced spectroscopic methods (^1^H/^13^C NMR and MS) to discover these compounds in distinct life-cycle stages, including plasmodia and fruiting bodies.

Ishibashi and co-workers [79] analyzed *Lindbladia tubulina* fruiting bodies, identifying 6,7-dimethoxydihydrolindbladione and 6,7-dimethoxylindbladione, both noted subsequently in comprehensive surveys [66]. In a related study, Hosoya et al. [30] reported 6-methoxydihydrolindbladione from *Perichaena chrysosperma* and *Lycogala epidendrum*, with further confirmation in *L. tubulina* [66,79]. Additional variants, such as 7-methoxylindbladione, appeared in *L. tubulina* [66,79]. Parallel work on *Cribraria* species demonstrated cribrariones A, B, and C, which Naoe et al. [99] and Iwata et al. [100] initially characterized as naphthoquinone derivatives with antimicrobial activities; subsequent efforts by Shintani [31] expanded this to include *Cribraria meylanii*, and reviews by Stoyneva-Gärtner et al. [66] have consolidated these findings.

In *Dictyostelium discoideum*, Saito et al. [90] uncovered two polyketide intermediates (desmethyl and desmethyl-monochloro analogues of DIF-1) that induce stalk cell differentiation in developmental mutants. Meanwhile, Kubohara and Kikuchi [91] documented chlorinated alkylresorcinols (monochasiols A–H) from *Dictyostelium monochasioides*, following earlier isolation studies by Kikuchi et al. [101]. A summary of these “other polyketides”, including their source taxa and references, is presented in Table 10.

### 3.5. Pigments

Pigments in *Eumycetozoa* serve as notable coloration agents with essential physiological roles, such as photoprotection, antioxidant defense, and chemical signaling. They range from carotenoids and melanins to a variety of bisindole and naphthoquinone derivatives, often arising via polyketide, isoprenoid, or amino-acid-based pathways. Their striking colors, most evident in plasmodial and fruiting body structures, underscore the ecological significance of these secondary metabolites in slime molds.

Early work on carotenoid pigments in *Dictyostelium discoideum* involved solvent extractions, saponification, and chromatographic purification, confirming that the bright yellow coloration of fruiting bodies stems from de novo synthesis of zeta-carotenes [102]. Later surveys, including broader reviews of *Myxogastria* secondary metabolism, corroborate the presence of acidic carotenoids and highlight their photoprotective capacities [56,58,66]. These studies generally employed methanol or acetone extraction, followed by partitioning in ethyl acetate and water, with subsequent fractionation on silica-based media and verification of structures by UV spectroscopy and MS.

Bisindole pigments have garnered particular attention in the genus *Arcyria*. Steglich and co-workers [78], for instance, reported isolations of arcyriacyanin A, arcyriarubin B, and arcyriarubin C from wild-collected *Arcyria denudata* fruiting bodies, using solvent partitioning and Sephadex LH-20 chromatography, then elucidating structures with ^1^H/^13^C NMR. More recent investigations further revealed compounds such as dihydroarcyriacyanin A [103,104] and dihydroarcyriarubin C [79,105] in *Arcyria ferruginea*, *Arcyria obvelata*, and *Tubifera casparyi*, typically applying similar extraction and fractionation strategies. Several of these bisindoles display cytotoxic or cell-cycle-inhibitory properties, confirmed through assays on human cancer cell lines.

Fuligorubin A, most notably found in the plasmodia of *Fuligo septica*, has undergone extensive characterization through chromatographic fractionation (e.g., silica gel and reversed-phase HPLC) and total synthetic approaches [95,106,107,108,109]. Review articles also affirm its presence and highlight potential ecological functions [56,58,66,78]. Melanin and melanin-like pigments have been described in darkened sclerotia of *Fuligo septica* [110] and in fruiting bodies of *Physarum nebulosum* [66], while naphthoquinones, including those isolated from *Cribraria purpurea* via solvent extraction and gel filtration [78,79], further diversify the myxomycete pigment repertoire. Physarochrome A, similarly characterized by advanced spectroscopy and hydrogenation studies [78,94], exemplifies yet another specialized pigment found in *Physarum* and *Trichia*. A concise overview of these and other pigments is provided in Table 11.

### 3.6. Alkaloids and Indole Derivatives

Alkaloids in slime molds (*Eumycetozoa*) encompass a remarkable range of nitrogen-containing heterocycles, frequently exhibiting significant biological activities such as cytotoxicity, antibiotic properties, and kinase inhibition. Many of these compounds feature indole or bisindole scaffolds—including staurosporine analogs, makaluvamines, and arcyriaflavins—that can function in chemical defense, developmental regulation, or adaptation to environmental stress. As summarized in Table 12, these alkaloids have been detected across various life stages (e.g., fruiting bodies vs. plasmodia) in multiple genera, often through systematic isolation and spectroscopic identification protocols.

Research efforts have consistently relied on similar methodologies for alkaloid extraction and characterization. Typical workflows begin with field collection of fruiting bodies in Japan (notably Kochi Prefecture) or other locales, followed by air-drying and exhaustive extraction using 90% methanol or acetone [76,82,97]. The crude extracts are then partitioned between ethyl acetate and water, and subsequently fractionated using silica gel, ODS (octadecyl silica), or Sephadex LH-20 chromatography [79,105]. Final purification steps often involve reversed-phase HPLC, yielding sufficient material for structural elucidation via ^1^H/^13^C NMR (including 2D experiments such as HMBC and HMQC), MS, and comparison with previously characterized compounds [56,58,66,78].

Several notable bisindole alkaloids have been reported in *Arcyria* species, including arcyriaflavins A–C [30,78,97], arcyroxindole A [78,112], and derivatives of staurosporine (e.g., 6-hydroxystaurosporinone) [30,76,79]. In *Arcyria obvelata* (*A. o.*), fruiting bodies have yielded arcyriaflavin B, arcyriaflavin C, and the colorless dihydro-forms of related compounds when subjected to repeated chromatographic steps [58,76,105,113]. Shintani [76] further documented 6-hydroxy-9’-methoxystaurosporinone in *Perichaena chrysosperma* (*P. c.*), identified via high-resolution MS and ^1^H/^13^C NMR, and showed its ability to inhibit hedgehog signaling in mammalian cell assays.

Other slime mold taxa also harbor diverse indole alkaloids. *Lycogala epidendrum* (*L. e.*) features 5,6-dihydroxyarcyriaflavin A [30,79] and staurosporinone [58,79], often isolated from methanol extracts of fruiting bodies and assessed for cytotoxic or protein kinase inhibitory actions. Likewise, *Lindbladia tubulina* (*L. t.*) is a known source of 6,9’-dihydroxystaurosporinone, which was first reported through multi-step fractionation and advanced spectroscopic methods [56,74]. Makaluvamines, including makaluvamine A, B, and I, have emerged from fruiting bodies or plasmodia of *Didymium* and *Lycogala* species [29,58,66,82,114], frequently demonstrating antibacterial or topoisomerase-inhibitory activity previously associated with marine-derived pyrroloiminoquinones.

Researchers have also documented simpler indole derivatives, such as *(Z)*-methyl-2-hydroxy-3-(1H-indol-3-yl)acrylate [58,78] and 3,4-bis(indol-3-yl)pyrrole-2,5-dicarboxylic acid [56,58,66], in fruiting bodies or plasmodia of several genera. These findings have often been corroborated by broader reviews, such as that of Dembitsky [56] and the more recent literature compilations by Li et al. [58] and Stoyneva-Gärtner et al. [66], underscoring the importance of consistent taxonomic verification, solvent extraction protocols, and the role of advanced analytical platforms in characterizing the structural diversity of slime mold alkaloids. Collectively, these studies highlight that *Eumycetozoa* alkaloids not only represent intriguing chemical scaffolds for natural product research but also reinforce the potential of slime molds as emerging sources of pharmacologically active compounds. A detailed overview of the reported alkaloids is presented in Table 12.

**Table 12:** Alkaloids and indole derivatives isolated from *Eumycetozoa*, arranged alphabetically. The table includes compound names, corresponding species, and the life-cycle stages where they have been identified.

Compound Name	Source *Eumycetozoa* Species	Life-Cycle Structure	References
***(Z)*****-methyl-2-hydroxy-3-(1H-indol-3-yl)acrylate**	*A. d.*	Fruiting body	[58,78]
**3,4-bis(indol-3-yl)pyrrole-2,5-dicarboxylic acid**	*P. p.*	Plasmodium	[56,58,66]
**5,6-Dihydroxyarcyriaflavin A**	*L. e.*	Fruiting body	[30,58,66,79]
**Arcyriaflavin A**	*L. e.*, *A. o.*	Fruiting body	[30,58,66,78,96,97]
**Arcyriaflavin B**	*A. o.*, *T. c.*, *L. e.*	Fruiting body	[58,66,76,78,79,82,97,105,113]
**Arcyriaflavin C**	*A. o.*, *M. v.*, *T. c.*	Fruiting body	[58,66,78,79,82,105]
**Arcyroxindole A**	*A. d.*, *M. v.*	Fruiting body	[58,66,78,112]
**6,9’-Dihydroxystaurosporinone**	*L. t.*	Fruiting body, Plasmodium	[56,58,66,74,76]
**6-Hydroxystaurosporinone**	*P. c.*, *L. e.*	Fruiting body	[30,66,76,79,113]
**6-Hydroxy-9’-methoxystaurosporinone**	*A. c.*, *P. c.*	Fruiting body	[58,76,113]
**Makaluvamine A**	*D. i., L. e., D. b.*	Fruiting body	[29,58,66,82]
**Makaluvamine B**	*L. e., D. b.*	Fruiting body	[29,66,82]
**Makaluvamine I**	*D. b.*, *D. i.*, *L. e.*	Plasmodium, Fruiting body	[29,58,79,114]
**Staurosporinone**	*L. e.*	Fruiting body	[58,79]

**Abbreviations:** A. c., *Arcyria cinerea*; A. d., *Arcyria denudata*; A. o., *Arcyria obvelata*; D. b., *Didymium bahiense*; D. i., *Didymium iridis*; L. e., *Lycogala epidendrum*; L. t., *Lindbladia tubulina*; M. v., *Metatrichia vesparium*; P. c., *Perichaena chrysosperma*; P. p., *Physarum polycephalum*; T. c., *Tubifera casparyi*.

### 3.7. Polyenes and Polyacetylenes

Polyenes and polyacetylenes are distinguished by their extended networks of unsaturated bonds—either conjugated double bonds or triple-bond (acetylenic) linkages. Such structural features often confer striking coloration, strong electron affinity, and a capacity for diverse bioactivities, including antifungal, antibiotic, or cytotoxic effects. In slime molds (*Eumycetozoa*), these molecules have been identified in fruiting bodies and plasmodia through a combination of solvent extraction, chromatographic fractionation, and advanced spectroscopic analyses, as outlined below and in Table 13.

A recurring approach involves collecting fruiting bodies from field sites (e.g., decaying wood) or cultivating plasmodia on agar plates supplemented with oats or bacterial prey. Subsequent extraction with methanol, acetone, or chloroform typically precedes partitioning into polar and non-polar phases. Many investigators then employ silica gel, ODS (octadecyl silica), or Sephadex LH-20 column chromatography to isolate individual fractions. Notable examples include the purification of ceratiopyrons A–D and ceratioflavin A from *Ceratiomyxa fruticulosa* [58,66,78,82], as well as cinereapyrroles A and B from *Arcyria cinerea* [66,79,97]. Spectral characterization using ^1^H/^13^C NMR (1D and 2D), MS, IR, and UV techniques then confirms structural details and, in some cases, provides insight into bioactivities such as cytotoxic or antibiotic properties [56,66,82].

In *Fuligo septica*, investigators have identified fuligoic acid and its dehydro-derivative through reversed-phase ODS chromatography, elucidating their polyene–pyrone cores by ^1^H/^13^C NMR and MS [58,66,115]. The same species also yields fuligopyrone A and B, reported by Steglich [78] and Minns [109], respectively, with evidence suggesting a protective role against UV-induced stress. Dictyostelid slime molds, including *Dictyostelium discoideum* and *D. firmibasis*, produce the dictyopyrones and dihydrodictyopyrones, characterized by Takaya [116], Kikuchi [117], and Kubohara [91]. Their findings, confirmed through HMBC and ^1^H/^13^C NMR experiments, indicate that subtle modifications in side-chain length or hydrogenation patterns can alter biological function. Further examples from *Dictyostelium* species include the bispyrone analogs and dictyomedins [58,66,118,119], which exhibit inhibitory effects on slime mold development or smooth muscle contraction.

Additional polyenes and polyacetylenes have been observed in *Lindbladia tubulina* (lindbladiapyrone) [56,66,78] and in *Tubulifera arachnoidea* (tubiferic acid) [58]. Across these studies, some compounds undergo total or partial synthesis to corroborate proposed structures (e.g., ceratiopyrons, dictyopyrones, and fuligopyrones), while others are screened for antimicrobial or cytotoxic potential against bacterial and cancer cell lines [78,109]. Comprehensive reviews by Li [58], Stoyneva-Gärtner [66], and Dembitsky [56] confirm many of these findings, emphasizing how variations in polyketide and fatty-acid-derived pathways underlie the chemical diversity of these conjugated metabolites in *Myxogastria*. A condensed overview of these polyenes and polyacetylenes, their source organisms, and their life-cycle contexts is presented in Table 13.

**Table 13:** Compilation of polyenes and polyacetylenes identified in *Eumycetozoa*. Listed in alphabetical order are the compounds, the source *Eumycetozoa* species, the life cycle structures in which they were found, and the corresponding references.

Compound Name	Source *Eumycetozoa* Species	Life-Cycle Structure	References
**Ceratioflavin A**	*C. f.*	Fruiting body	[82]
**Ceratiopyron A**	*C. f.*	Fruiting body	[58,66,78,82]
**Ceratiopyron B**	*C. f.*	Fruiting body	[82]
**Ceratiopyron C**	*C. f.*	Fruiting body	[82]
**Ceratiopyron D**	*C. f.*	Fruiting body	[82]
**Cinereapyrrole A**	*A. c.*	Fruiting body	[66,79,97]
**Cinereapyrrole B**	*A. c.*	Fruiting body	[66,79,97]
**Dehydrofuligoic acid (chlorinated polyene-pyrone acid)**	*F. s.*	Fruiting body	[115]
**Dictyobispyrone B**	*D. g.*	Fruiting body	[66,91,119]
**Dictyobispyrone E**	*D. g.*	Fruiting body	[66,91,119]
**Dictyomedin A**	*D. d.*	Fruiting body	[58,66,118]
**Dictyomedin B**	*D. d.*	Fruiting body	[58,118]
**Dictyopyrone A**	*D. d., D. r., D. l.*	Fruiting body, Cellular stage	[58,66,91,116]
**Dictyopyrone B**	*D. d., D. r., D. l., C. f.*	Fruiting body, Cellular stage, Plasmodium	[58,66,78,91,116]
**Dictyopyrone C**	*D. d., D. r., D. l.*	Fruiting body, Cellular stage	[58,91,116]
**Dictyopyrone D**	*D. d., D. r., D. l.*	Fruiting body, Cellular stage	[91]
**Dihydrodictyopyrone A**	*D. f.*	Fruiting body	[66,91,117]
**Dihydrodictyopyrone C**	*D. f.*	Fruiting body	[66,91,117]
**Fuligoic Acid**	*F. s.*	Fruiting body	[58,66,115]
**Fuligopyrone A**	*F. s., F. c.*	Plasmodium	[58,66,78,109]
**Fuligopyrone B**	*F. s.*	Fruiting body	[109]
**Lindbladiapyrone**	*L. t., T. f., D. i.*	Fruiting body, Plasmodium	[56,66,78]
**Tubiferic Acid**	*T. a.*	Fruiting body	[58]

**Abbreviations:** A. c., *Arcyria cinerea*; C. f., *Ceratiomyxa fruticulosa*; D. d., *Dictyostelium discoideum*; D. f., *Dictyostelium firmibasis*; D. g., *Dictyostelium giganteum*; D. i., *Didymium iridis*; D. l., *Dictyostelium longosporum*; D. r., *Dictyostelium rhizoposium*; F. c., *Fuligo cinerea*; F. s., *Fuligo septica*; L. t., *Lindbladia tubulina*; T. a., *Tubulifera arachnoidea*; T. f., *Trichia floriformis*.

### 3.8. Other Eumycetozoa-Specific Secondary Metabolites

Other *Eumycetozoa*-specific secondary metabolites encompass a broad spectrum of chemically distinct entities not readily assignable to the previously covered classes (e.g., pigments, terpenoids, or alkaloids). This collective includes acyltetramic acids, various heterocyclic antibiotics, unusual sugar derivatives, polyketide-based dibenzofurans, lectin-like proteins, and other rare scaffolds. Despite their disparate origins and structural diversity, these compounds often exhibit notable bioactivities, such as antimicrobial, cytotoxic, or cell-differentiation effects, and sometimes appear restricted to a particular slime mold taxon or developmental stage. Table 14 provides a consolidated overview of these specialized metabolites, their source species, and the pertinent literature.

Several studies have employed similar workflows to isolate these compounds from fruiting bodies, plasmodia, or myxamoebae cultured on nutrient agar containing bacterial prey. Typical procedures involve solvent extraction with methanol, acetone, or chloroform, followed by partitioning into polar and non-polar phases. The resulting fractions are then subjected to silica gel or ODS (octadecyl silica) column chromatography, Sephadex LH-20 gel filtration, and, in many cases, reversed-phase HPLC for final purification. Characterization generally relies on 1D and 2D ^1^H/^13^C NMR analyses, MS, and, when necessary, single-crystal X-ray diffraction or total synthesis to validate proposed structures.

Distinct benzene-1,3-diol analogs, such as EPBD (4-ethyl-5-pentylbenzene-1,3-diol) and PPBD (4-*n*-propyl-5-pentylbenzene-1,3-diol), were synthesized and biologically evaluated by Murata [92] after their identification in the cellular stage of *Dictyostelium discoideum*. Other noteworthy examples include acyltetramic acids in *Leocarpus fragilis* [66,78] and anthraquinonic acids from plasmodia of *Fuligo septica* [32], both isolated via repeated solvent extraction and subsequent chromatographic fractionation. In *Lycogala epidendrum*, Fröde [96] described the methanolic extraction of fruiting bodies that led to the identification of amyriarubin A, whereas *Arcyria denudata* has yielded compounds such as arcyrioxepin A and B [32,66,77], arcyriaverdin C [78], and arcyrioxocin A and B [66,78] through successive chromatography and ^1^H/^13^C NMR-based structural elucidation.

Research on *Arcyria* and *Tubifera* has likewise revealed arcyroxepins A and B in *Lycogala epidendrum* [58,77,78] and arcyroxocins A and B in *Arcyria denudata* and *Tubifera casparyi* [58,66,104,105,112,120]. Other unique molecules, including bahiensol in *Didymium bahiense* and *Polysphondylium pallidum* [58,66,121], and brefelamide in *Dictyostelium bahiense* and *D. giganteum* [66,91,122], have surfaced through similar fractionation protocols, often followed by spectroscopic confirmation (1D/2D ^1^H/^13^C NMR and MS).

In the genus *Didymium*, damirone C stands out for having been detected in *Didymium iridis* and *Lycogala epidendrum*, with isolation and partial synthesis detailed by Ishibashi [79], Nakatani [114], and others [58,66]. Similarly, melleumin A and B, originally found in *Physarella melleum* and subsequently in *Arcyria denudata*, were extracted from both plasmodia and fruiting bodies by Nakatani [123] and Ishibashi [79], who confirmed structures using advanced ^1^H/^13^C NMR methods and bioassays for antimicrobial or cytotoxic activities [58,66,78]. Additional advanced glycosides, such as dictyoglucosamine A and B in *Dictyostelium purpureum* and *D. discoideum* and furanodictine A and B in *D. discoideum*, were elucidated by Kikuchi [80,83,124] and highlighted in subsequent reviews [58,91] for their neurite-outgrowth-promoting properties.

Among antibiotic-like factors, Schroeder and Mallette [33] first isolated D-1 (a heterocyclic antibiotic fraction) from plasmodia of *Physarum gyrosum*, while Tafakori [32] later discussed this and related antimicrobial compounds in a broader context. Enteridinines A and B, discovered in *Enteridium lycoperdon* and *Reticularia lycoperdon* by Řezanka and co-workers [66,125,126], likewise emerged through multi-step solvent extractions and gel filtration, showing inhibitory effects on bacterial growth. Kehokorins A–E, a family of dibenzofuran derivatives isolated from *Trichia favoginea*, have been investigated by Kaniwa [127], Ishibashi [128], Takahashi [129], and Li [58] for their cytotoxic activities. Additional discoidin-type proteins, exemplified by the hemagglutinin physarumin in *Physarum polycephalum*, were purified by ammonium sulfate precipitation and multiple chromatographic steps, then characterized for Ca^2+^-dependent lectin activity [130].

Particularly noteworthy among *Dictyostelium* taxa are the differentiation-inducing factor DIF-1 and its derivatives [91,130], which modulate stalk-cell formation and display antitumor and immunoregulatory properties. Several specialized polyketide-derived compounds—such as the benzene diols MPBD, EPBD, and PPBD—were likewise reported in *D. discoideum*, after synthetic studies and structure–activity relationship assays [92]. Taken together, these findings underscore the intricate chemical repertoire of slime molds and the wide-ranging methodological approaches—encompassing field collection, in-lab cultivation, extensive chromatographic fractionation, spectral analyses, and synthetic confirmation—applied to uncover structurally unique metabolites. A complete synopsis of these specialized substances, their life-cycle phases, and relevant primary sources is presented in Table 14.

**Table 14:** Specialized or unique secondary metabolites from *Eumycetozoa* that do not clearly fit into preceding classes. Listed alphabetically are the compound names, the species of origin, the corresponding life-cycle structures, and the primary literature.

Compound Name	Source *Eumycetozoa* Species	Life Cycle Structure	References
**4-ethyl-5-pentylbenzene-1,3-diol (EPBD)**	*D. d.*	Cellular stage	[92]
**4-n-propyl-5-pentylbenzene-1,3-diol (PPBD)**	*D. d.*	Cellular stage	[92]
**Acyltetramic acids**	*L. f.*	Plasmodium	[66,78]
**Amyriarubin A**	*L. e.*	Fruiting body	[96]
**Anthraquinonic acids**	*F. s.*	Plasmodium	[32]
**Arcyrioxepin A**	*A. d.*	Fruiting body	[32,66,77]
**Arcyrioxepin B**	*A. d.*	Fruiting body	[32,66]
**Arcyriaverdin C**	*A. d.*	Fruiting body	[78]
**Arcyrioxocin A**	*A. n., A. d.*	Fruiting body	[66,78]
**Arcyrioxocin B**	*A. n., A. d.*	Fruiting body	[66,78]
**Arcyroxepin A**	*L. e.*	Fruiting body	[58,77,78]
**Arcyroxepin B**	*L. e.*	Fruiting body	[58,77,78]
**Arcyroxocin A**	*A. d., T. c.*	Fruiting body	[58,66,105,112,120]
**Arcyroxocin B**	*A. d., T. c.*	Fruiting body	[58,66,104,112,120]
**Bahiensol**	*D. b., P. p.*	Plasmodium, Myxamoebae	[58,66,121]
**Brefelamide**	*D. b., D. g.*	Fruiting body	[66,91,122]
**D-1 (antibiotic fraction)**	*P. g.*	Plasmodium	[33]
**Damirone C**	*D. i., L. e.*	Plasmodium, Fruiting body	[58,66,79,114]
**Dictyoglucosamine A**	*D. p.*	Fruiting body	[58,80,83,91]
**Dictyoglucosamine B**	*D. d.*	Fruiting body	[58,80,83,91]
**DIF-1**	*D. d.*	Fruiting body	[91,130]
**DIF-1 derivatives**	*D. d.*	Fruiting body	[91,130]
**Enteridinines A**	*E. l., R. l.*	Plasmodium	[66,125,126]
**Enteridinines B**	*E. l., R. l.*	Plasmodium	[66,125,126]
**Fulicineroside**	*F. c.*	Plasmodium	[58,66,131]
**Furanodictine A**	*D. d.*	Fruiting body	[58,83,91,124]
**Furanodictine B**	*D. d.*	Fruiting body	[58,83,91,124]
**Heterocyclic antibiotic D-1**	*P. g.*	Plasmodium	[32,33]
**Kehokorin A**	*T. f.*	Fruiting body	[58,127,129]
**Kehokorin B**	*T. f.*	Fruiting body	[58,127,129]
**Kehokorin C**	*T. f.*	Fruiting body	[58,127,129]
**Kehokorin D**	*T. f.*	Fruiting body	[128,129]
**Kehokorin E**	*T. f.*	Fruiting body	[128,129]
**Lycogaride A**	*D. b.*	Plasmodium	[58,132]
**Lycogaride B**	*D. b.*	Plasmodium	[58,132]
**Lycogaride C**	*D. b., L. e.*	Plasmodium, Fruiting body	[58,132]
**Lycogaride D**	*L. e.*	Fruiting body	[29,58,98]
**Lycogaride E**	*L. e.*	Fruiting body	[29,58,98]
**Lycogaride F**	*L. e.*	Fruiting body	[29,58,98]
**Lycogaride G**	*L. e.*	Fruiting body	[29,58,98]
**Melleumin A**	*P. m., A. d.*	Fruiting body, Plasmodium	[58,66,78,79,123]
**Melleumin B**	*P. m., A. d.*	Fruiting body, Plasmodium	[58,78,79,123]
**Physarigin A**	*P. p., P. r.*	Fruiting body	[28,58,66,79]
**Physarigin B**	*P. p., P. r.*	Fruiting body	[28,58,66,79]
**Physarigin C**	*P. p., P. r.*	Fruiting body	[28,58,66,79]

**Abbreviations:** A. d., *Arcyria denudata*; A. n., *Arcyria nutans*; D. b., *Didymium bahiense*; D. d., *Dictyostelium discoideum*; D. g., *Dictyostelium giganteum*; D. i., *Didymium iridis*; D. p., *Didymium purpureum*; E. l., *Enteridium lycoperdon*; F. c., *Fuligo cinerea*; F. s., *Fuligo septica*; L. e., *Lycogala epidendrum*; L. f., *Leocarpus fragilis*; P. g., *Physarum gyrosum*; P. m., *Physarella millecaput*; P. p., *Polysphondylium pallidum*; P. r., *Physarum rigidum*; R. l., *Reticularia lycoperdon*; T. c., *Tubifera casparyi*; T. f., *Trichia favoginea*.

## 4. Summary and Conclusions

In this review, we surveyed a total of 298 distinct chemical substances produced by true slime molds (*Eumycetozoa*), covering carbohydrates, amino acids, proteins, lipids (including saturated and unsaturated fatty acids, phospholipids, and sterols), pigments, alkaloids, polyketides, and a wide range of other specialized secondary metabolites. These compounds appear to be closely tied to developmental stages (e.g., plasmodial feeding, sporulation, and fruiting-body formation) and ecological functions (e.g., photoprotection and antimicrobial defense). Although various biological roles—particularly antimicrobial, cytotoxic, or signaling-related—have been elucidated in biomedical or fundamental research contexts, their applications in forestry and agriculture remain comparatively understudied.

Carbohydrate-based molecules such as lycogalinosides A and B (identified in *Lycogala epidendrum* and *Dictyostelium rhizoposium*) selectively inhibit Gram-positive bacteria [55,56,57,58], suggesting relevance for controlling nursery pathogens. Lectins such as discoidin (from *Dictyostelium discoideum*) and physarumin (from *Physarum polycephalum*) may be repurposed to disrupt microbial cell walls or impede pathogen attachment, as demonstrated in other microbial eukaryotes [67]. Free fatty acids and polyunsaturated fatty acids (PUFAs) synthesized by slime molds (e.g., arachidonic and eicosapentaenoic acids) similarly warrant investigation, since related lipids can suppress fungal spore germination and viability [56,66,70].

Polyketide-derived compounds also exhibit a broad spectrum of bioactivities. Staurosporine analogs (e.g., 6-hydroxystaurosporinone) and makaluvamines can inhibit fungal growth or moderate bacterial proliferation [76,79], whereas quinones (e.g., cribrariones and lindbladiones) and bisindole polyketides (e.g., lycogarubins) could potentially control pathogens such as *Botrytis*, *Fusarium*, or *Phytophthora* [30,74,99,100]. Sterols and terpenoids (e.g., tubiferal A/B, mucoroidiol, stigmasterol, and β-sitosterol) further diversify the spectrum of potential antifungal or insecticidal agents, given their structural parallels with well-known phytochemicals [56,87,88].

In Table 15, the major classes of slime mold metabolites are organized according to their documented or putative modes of action relevant to pest and pathogen management. Despite strong indications of antimicrobial and insecticidal potential, relatively few of these compounds have been rigorously evaluated under in vitro or in vivo conditions that mimic practical forestry or agricultural challenges. Insights derived from biomedical and basic research contexts can guide systematic assays, field trials, and toxicity profiling to determine whether slime mold metabolites can be deployed safely and effectively at scale. Continued biochemical, genomic, and ecological studies of *Eumycetozoa* will be essential for uncovering additional metabolites, clarifying biosynthetic pathways, and understanding the genetic or environmental cues governing compound production. By leveraging structure–activity relationship analyses and targeted biocontrol assays, these investigations may pave the way for harnessing the chemical diversity of true slime molds as a sustainable resource in pest and disease control strategies.

The authors also intend to conduct further biochemical assays and genomic analyses to enhance the understanding of the evolutionary significance and functional diversity of *Eumycetozoa*. Specifically, future studies will focus on the biochemical characterization of extracts obtained from *Lycogala epidendrum* during its plasmodial and fruiting body stages, sourced from various forested regions. Recognizing the chemical potential of slime molds underscores their ecological and industrial importance, thereby facilitating the discovery of novel applications. A comprehensive investigation of their unique metabolomes may identify naturally occurring pesticides and fungicides that, upon validation, could serve as eco-friendly alternatives to conventional agrochemicals. Such advancements would contribute to the development of integrated pest management strategies, bolster the resilience of forest and agricultural systems against pathogens and pests, and expand the horizons of microbial natural product research.

## Figures and Tables

**Table 1:** Carbohydrates, their derivatives, and glycoconjugates isolated from *Eumycetozoa*. The table lists compound names, source species, life-cycle structures, and the corresponding references.

Compound Name	Source *Eumycetozoa* Species	Life-Cycle Structure	References
**β-** ***D*** **-galactan**	*P. p.*	Plasmodium	[53]
**Fucose**	*P. p.*	Plasmodium	[54]
**Galactose**	*P. f.*, *P. p.*, *P. o.*, *P. r.*, *D. d.*	Slime track, Fruiting body	[49,50,51]
**Galacturonic acid**	*D. d.*	Fruiting body	[51]
**Glucosamine**	*P. p.*, *D. d.*	Fruiting body, Plasmodium	[51,54]
**Glucose**	*P. p.*, *P. o.*, *D. d.*	Slime track, Fruiting body, Plasmodium	[50,51,54]
**Glucuronic acid**	*D. d.*	Fruiting body	[51]
**Lycogalinoside A** (2-deoxy-α-*l*-fucopyranosyl-(1→4)-6-deoxy-β-*d*-gulopyranoside of lycogaline)	*L. e.*, *D. r.*	Fruiting body	[55,56,57,58]
**Lycogalinoside B** (β-*d*-olivopyranosyl-(1→4)-β-*d*-fucopyranoside)	*L. e.*, *D. r.*	Fruiting body	[55,56,57,58]
**Mannose**	*P. p.*, *D. d.*	Fruiting body, Plasmodium	[51,54]
**Rhamnose**	*P. p.*, *P. o.*	Slime track	[50]
**Trehalose**	*D. d.*	Slime track	[52]

**Abbreviations:** D. d., *Dictyostelium discoideum*; D. r., *Dictyostelium rhizoposium*; L. e., *Lycogala epidendrum*; P. f., *Physarum flavicomun*; P. o., *Physarella oblonga*; P. p., *Physarum polycephalum*; P. r., *Physarum rigidum*.

**Table 2:** Proteinogenic and non-proteinogenic amino acids reported in various *Eumycetozoa*, including the original source species, developmental stages, and key references.

Compound Name	Source *Eumycetozoa* Species	Life-Cycle Structure	References
**Alanine**	*D. d.*, *P. p.*	Spores, Microcysts	[59,60]
**Aspartic acid**	*D. d.*, *P. p.*	Spores, Microcysts	[59,60]
**Glutamic acid**	*D. d.*, *P. p.*	Spores, Microcysts	[59,60]
**Glycine**	*D. d.*, *P. p.*	Spores, Microcysts	[59,60]
**Lysine**	*D. d.*, *P. p.*	Spores, Microcysts	[59,60]
**Serine**	*D. d.*, *P. p.*	Spores, Microcysts	[59,60]
**Threonine**	*D. d.*, *P. p.*	Spores, Microcysts	[59,60]
**Arginine**	*D. d.*, *P. p.*	Spores, Microcysts	[59,60]
**Histidine**	*D. d.*, *P. p.*	Spores, Microcysts	[59,60]
**Isoleucine**	*D. d.*, *P. p.*	Spores, Microcysts	[59,60]
**Leucine**	*D. d.*, *P. p.*	Spores, Microcysts	[59,60]
**Methionine**	*D. d.*, *P. p.*	Spores, Microcysts	[59,60]
**Phenylalanine**	*D. d.*, *P. p.*	Spores, Microcysts	[59,60]
**Proline**	*D. d.*, *P. p.*	Spores, Microcysts	[59]
**Tyrosine**	*D. d.*, *P. p.*	Spores, Microcysts	[59,60]
**Valine**	*D. d.*, *P. p.*	Spores, Microcysts	[59,60]

**Abbreviations:** D. d., *Dictyostelium discoideum*; P. p., *Physarum polycephalum*.

**Table 3:** Enzymes, peptides, and other macromolecular proteins reported in *Eumycetozoa*. The table includes compound/protein names, source species, typical life-cycle structure, and references.

Compound Name	Source *Eumycetozoa* Species	Life-Cycle Structure	References
**Actin**	*D. d.*	Plasmodium	[62]
**Alanine aminotransferase (ALT)**	*D. d.*	Plasmodium	[61]
**Aminopeptidase**	*D. d.*	Plasmodium	[61]
**Aspartate aminotransferase (AST)**	*D. d.*	Plasmodium	[61]
**Delta-5-fatty acid desaturase (enzyme activity)**	*D. d.*	Fruiting body	[65,66]
**Discoidin (Lectin/adhesion protein)**	*D. d.*	Plasmodium	[67]
**Glorin**	*P. v.*	Fruiting body	[68]
**Glutamate dehydrogenase**	*D. d.*	Plasmodium	[61]
**Lactate dehydrogenase**	*D. d.*	Fruiting body	[61]
**Myosin II**	*D. d.*	Fruiting body	[63,64,66]
**Pallidin**	*P. p.*	Plasmodium	[67]
**Physarumin (Hemagglutinin)**	*P. p.*	Plasmodium	[67]

**Abbreviations:** D. d., *Dictyostelium discoideum*; P. p., *Physarum polycephalum*; P. v., *Polysphondylium violaceum.*

**Table 4:** Saturated and branched-chain fatty acids documented in *Eumycetozoa*. Standard nomenclature is consistently provided for each acid. Compounds are listed alphabetically by their trivial or structural name.

Compound Name	Source *Eumycetozoa* Species	Life-Cycle Structure	References
**10-Methyldodecanoic acid**	*A. c.*, *A. d.*, *A. n.*, *F. s.*, *L. e.*, *L. f.*, *P. sp.*, *T. f.*, *T. v.*	Fruiting body	[56,70]
**12-Methyltetradecanoic acid**	*A. c.*, *A. d.*, *A. n.*, *F. s.*, *L. e.*, *L. f.*, *P. sp.*, *T. f.*, *T. v.*	Fruiting body	[56,70]
**13-Methylpentadecanoic acid**	*P. p.*	Plasmodium	[71]
**13-Methyltetradecanoic acid**	*A. c.*, *A. d.*, *A. n.*, *L. e.*, *L. f.*, *T. f.*, *T. v.*	Fruiting body	[56,70]
**14-Methylhexadecanoic acid**	*F. s.*, *P. sp.*	Fruiting body	[56,70]
**14-Methylpentadecanoic acid**	*A. c.*, *A. d.*, *A. n.*, *F. s.*, *L. e.*, *L. f.*, *P. sp.*, *T. f.*, *T. v.*	Fruiting body	[56,70]
**15-Methylhexadecanoic acid**	*A. c.*, *A. d.*, *A. n.*, *L. e.*, *L. f.*, *T. f.*, *T. v.*	Fruiting body	[56,70]
**16-Methylheptadecanoic acid**	*A. c.*, *A. d.*, *A. n.*, *L. e.*, *L. f.*, *T. f.*, *T. v.*	Fruiting body	[56,70]
**16-Methyloctadecanoic acid**	*A. c.*, *A. d.*, *A. n.*, *L. e.*, *L. f.*, *T. f.*, *T. v.*	Fruiting body	[56,70]
**17-Methyloctadecanoic acid**	*A. c.*, *A. d.*, *A. n.*, *L. e.*, *L. f.*, *T. f.*, *T. v.*	Fruiting body	[56,70]
**Arachidic acid (C_20_:0)**	*T. f.*, *T. v.*	Fruiting body	[56,69,70]
**Behenic acid (C_22_:0)**	*T. f.*, *T. v.*	Fruiting body	[56,70]
**Heptadecanoic acid (C_17_:0)**	*A. c.*, *A. d.*, *A. n.*, *F. s.*, *L. e.*, *L. f.*, *P. sp.*, *T. f.*, *T. v.*	Fruiting body	[56,70]
**Isoheptadecanoic acid**	*A. c.*, *A. d.*, *A. n.*, *F. s.*, *L. e.*, *L. f.*, *P. sp.*, *T. f.*, *T. v.*	Fruiting body	[56,70]
**Isohexadecanoic acid**	*P. p.*	Plasmodium	[71]
**Isononadecanoic acid**	*A. c.*, *A. d.*, *A. n.*, *L. e.*, *L. f.*, *T. f.*, *T. v.*	Fruiting body	[56,70]
**Isooctadecanoic acid, 1,1’-(1-methyl-1,2-ethanediyl) ester**	*A. c.*, *A. d.*, *A. n.*, *L. e.*, *L. f.*, *T. f.*, *T. v.*	Fruiting body	[56,70]
**Isopentadecanoic acid**	*F. s.*, *P. sp.*	Fruiting body	[56,70]
**Isotetradecanoic acid, TMS derivative**	*A. c.*, *A. d.*, *A. n.*, *F. s.*, *L. e.*, *L. f.*, *P. sp.*, *T. f.*, *T. v.*	Fruiting body	[56,70,71]
**Isotridecanoic acid**	*F. s.*, *L. f.*, *P. sp.*	Fruiting body	[56,70]
**Lauric acid (C_12_:0)**	*D. d.*	Fruiting body	[69]
**Lignoceric acid (C_24_:0)**	*A. c.*, *A. d.*, *A. n.*, *F. s.*, *L. e.*, *L. f.*, *P. sp.*, *T. f.*, *T. v.*	Fruiting body	[56,70]
**Myristic acid (C_14_:0)**	*A. c.*, *A. d.*, *A. n.*, *L. e.*, *L. f.*, *T. f.*, *T. v.*	Fruiting body	[56,69,70,72]
**Nonadecanoic acid (C_19_:0)**	*A. c.*, *A. d.*, *A. n.*, *L. e.*, *L. f.*, *T. f.*, *T. v.*	Fruiting body	[56,69]
**Palmitic acid (C_16_:0)**	*A. c.*, *A. d.*, *A. n.*, *L. e.*, *L. f.*, *T. f.*, *T. v.*	Fruiting body	[56,69,70,72,73]
**Pentadecanoic acid (C_15_:0)**	*A. c.*, *A. d.*, *A. n.*, *F. s.*, *L. e.*, *L. f.*, *P. sp.*, *T. f.*, *T. v.*	Fruiting body	[56,70]
**Stearic acid (C_18_:0)**	*A. c.*, *A. d.*, *A. n.*, *F. s.*, *L. e.*, *L. f.*, *P. sp.*, *T. f.*, *T. v.*	Fruiting body	[56,69,72]
**Tridecanoic acid (C_13_:0)**	*A. c.*, *A. d.*, *A. n.*, *F. s.*, *L. e.*, *L. f.*, *P. sp.*, *T. f.*, *T. v.*	Fruiting body	[56,70]

**Abbreviations:** A. c., *Arcyria cinerea*; A. d., *Arcyria denudata*; A. n., *Arcyria nutans*; D. d., *Dictyostelium discoideum*; F. s., *Fuligo septica*; L. e., *Lycogala epidendrum*; L. f., *Lycogala flavoscum*; P. p., *Physarum polycephalum*; P. sp., *Physarum species*; T. f., *Trichia favogiena*; T. v., *Trichia varia*.

**Table 5:** Summary of unsaturated fatty acids detected in *Eumycetozoa*. The table provides the alphabetical listing of compounds (using systematic/trivial names where relevant), species of origin, the life-cycle stage examined, and the associated primary literature.

Compound Name	Source *Eumycetozoa* Species	Life-Cycle Structure	References
**(9Z)-Heneicosenoic acid (21:1)**	*A. c.*, *A. d.*, *A. n.*, *F. s.*, *L. e.*, *L. f.*, *P. sp.*, *T. f.*, *T. v.*	Fruiting body	[56,70]
**(13Z)-Heneicosenoic acid**	*A. c.*, *A. d.*, *A. n.*, *F. s.*, *L. e.*, *L. f.*, *P. sp.*, *T. f.*, *T. v.*	Fruiting body	[56,70]
**(Z)-5-Hexadecenoic acid (C_16_:1,** Δ5 **)**	*A. c.*, *A. d.*, *A. n.*, *F. s.*, *L. e.*, *L. f.*, *P. sp.*, *T. f.*, *T. v.*	Fruiting body	[56,70]
**(Z)-7-Hexadecenoic acid (C_16_:1,** Δ7 **)**	*A. c.*, *A. d.*, *A. n.*, *F. s.*, *L. e.*, *L. f.*, *P. sp.*, *T. f.*, *T. v.*	Fruiting body	[56,70]
**5,8,11,14-Eicosatetraenoic acid (5,8,11,14–20:4)**	*A. c.*, *A. d.*, *A. n.*, *F. s.*, *L. e.*, *L. f.*, *P. sp.*, *T. f.*, *T. v.*	Fruiting body	[56,70]
**5,8,11,14–20:3 (8,11,14-Eicosatrienoic acid)**	*A. c.*, *A. d.*, *A. n.*, *F. s.*, *L. e.*, *L. f.*, *P. sp.*, *T. f.*, *T. v.*	Fruiting body	[56,72]
**5,9-Heptadecadienoic acid**	*D. d.*	Fruiting body	[69]
**5,9-Hexadecadienoic acid**	*L. e.*, *T. f.*, *T. v.*	Fruiting body	[30,56,69,70,72]
**5,9-Octadecadienoic acid (5,9–18:2)**	*T. f.*, *T. v.*	Fruiting body	[56,69,70,72]
**5,11-Octadecadienoic acid (5,11–18:2)**	*T. f.*, *T. v.*	Fruiting body	[56,69]
**5,11,14-Eicosatrienoic acid (5,11,14–20:3)**	*A. c.*, *A. d.*, *A. n.*, *F. s.*, *L. e.*, *L. f.*, *P. sp.*, *T. f.*, *T. v.*	Fruiting body	[56,70]
**5Z-Docosenoic acid**	*A. c.*, *A. d.*, *A. n.*, *F. s.*, *L. f.*, *P. sp.*, *T. v.*	Fruiting body	[56,70]
**5Z-Eicosenoic acid (5–20:1)**	*A. c.*, *A. d.*, *A. n.*, *F. s.*, *L. e.*, *L. f.*, *P. sp.*, *T. f.*, *T. v.*	Fruiting body	[56,70]
**5Z,13Z-Eicosadienoic acid (5,13–20:2)**	*T. f.*, *T. v.*	Fruiting body	[56,70]
**6,9,12-Octadecatrienoic acid**	*A. c.*, *A. d.*, *A. n.*, *F. s.*, *L. e.*, *L. f.*, *P. sp.*, *T. f.*, *T. v.*	Fruiting body	[56,72]
**6Z,9Z-Eicosadienoic acid (6,9–20:2)**	*A. c.*, *A. d.*, *A. n.*, *F. s.*, *L. e.*, *L. f.*, *P. sp.*, *T. f.*, *T. v.*	Fruiting body	[56,70]
**7,13-Docosadienoic acid**	*L. t.*	Fruiting body	[70,74]
**7,15-Docosadienoic acid**	*L. t.*	Fruiting body	[70,74]
**7Z-Docosenoic acid (C_22_:1,** Δ7 **)**	*A. c.*, *A. d.*, *A. n.*, *F. s.*, *L. f.*, *P. sp.*, *T. v.*	Fruiting body	[56,70]
**7Z-Eicosenoic acid (7–20:1)**	*A. c.*, *A. d.*, *A. n.*, *F. s.*, *L. e.*, *L. f.*, *P. sp.*, *T. f.*, *T. v.*	Fruiting body	[56,70]
**8,11,14–20:3 (8,11,14-Eicosatrienoic acid)**	*A. c.*, *A. d.*, *A. n.*, *F. s.*, *L. e.*, *L. f.*, *P. sp.*, *T. f.*, *T. v.*	Fruiting body	[56,72]
**8-Nonadecenoic acid (C_19_:1,** Δ8 **)**	*A. c.*, *A. d.*, *A. n.*, *F. s.*, *L. e.*, *L. f.*, *P. sp.*, *T. f.*, *T. v.*	Fruiting body	[56,70]
**8Z,11Z,14Z,17Z-Eicosatetraenoic acid (C_20_:4,** Δ8,11,14,17 **)**	*A. c.*, *A. d.*, *A. n.*, *F. s.*, *L. e.*, *L. f.*, *P. sp.*, *T. f.*, *T. v.*	Fruiting body	[56,70,72]
**9-Heptadecenoic acid**	*D. d.*	Fruiting body	[56,70]
**9Z-Octadecenoic acid (Oleic acid)**	*A. c.*, *A. d.*, *A. n.*, *F. s.*, *L. e.*, *L. f.*, *P. sp.*, *T. f.*, *T. v.*	Fruiting body	[56,70]
**9Z,12Z-Eicosadienoic acid (9,12–20:2)**	*A. c.*, *A. d.*, *A. n.*, *F. s.*, *L. e.*, *L. f.*, *P. sp.*, *T. f.*, *T. v.*	Fruiting body	[56,70]
**11Z-Octadecenoic acid (11–18:1)**	*A. c.*, *A. d.*, *A. n.*, *F. s.*, *L. e.*, *L. f.*, *P. sp.*, *T. f.*, *T. v.*	Fruiting body	[56,70]
**13-Docosenoic acid (C_22_:1,** Δ **13)**	*F. s.*, *P. sp.*	Fruiting body	[56,70]
**13–22:1 (Erucic acid)**	*A. c.*, *A. d.*, *A. n.*, *F. s.*, *L. e.*, *L. f.*, *P. sp.*, *T. f.*, *T. v.*	Fruiting body	[56,70]
**15Z-Docosenoic acid (C_22_:1,** Δ **15)**	*F. s.*, *P. sp.*	Fruiting body	[56,70]
**Adrenic acid (C_22_:4, n-6)**	*F. c.*	Fruiting body	[56,70]
**Alpha-linolenic acid (ALA, 9,12,15–18:3)**	*A. c.*, *A. d.*, *A. n.*, *F. s.*, *L. e.*, *L. f.*, *P. sp.*, *T. f.*, *T. v.*	Fruiting body	[56,70]
**Arachidonic acid (C_20_:4)**	*A. c.*, *A. d.*, *A. n.*, *F. s.*, *L. e.*, *L. f.*, *P. sp.*, *T. f.*, *T. v.*	Fruiting body	[70,72]
**Cetoleic acid (11Z-Eicosenoic acid)**	*A. c.*, *A. d.*, *A. n.*, *F. s.*, *L. e.*, *L. f.*, *P. sp.*, *T. f.*, *T. v.*	Fruiting body	[56,70,72]
**cis-8-Heptadecenoic acid**	*A. c.*, *A. d.*, *A. n.*, *F. s.*, *L. e.*, *L. f.*, *P. sp.*, *T. f.*, *T. v.*	Fruiting body	[56,70]
**cis-9-Heptadecenoic acid**	*A. c.*, *A. d.*, *A. n.*, *F. s.*, *L. e.*, *L. f.*, *P. sp.*, *T. f.*, *T. v.*	Fruiting body	[56,70]
**cis-9-Nonadecenoic acid**	*L. f.*, *P. sp.*	Fruiting body	[56,70]
**cis-10-Nonadecenoic acid (C_19_:1,** Δ10 **)**	*A. c.*, *A. d.*, *A. n.*, *F. s.*, *L. e.*, *L. f.*, *P. sp.*, *T. f.*, *T. v.*	Fruiting body	[56,70]
**cis-10-Nonadecenoic acid**	*A. c.*, *A. d.*, *A. n.*, *F. s.*, *L. e.*, *L. f.*, *P. sp.*, *T. f.*, *T. v.*	Fruiting body	[56,70]
**cis-11-Hexadecenoic acid**	*A. c.*, *A. d.*, *A. n.*, *F. s.*, *L. e.*, *L. f.*, *P. sp.*, *T. f.*, *T. v.*	Fruiting body	[70]
**cis-11,14-Eicosadienoic acid (11,14–20:2)**	*T. f.*, *T. v.*	Fruiting body	[56,70,72]
**cis-Vaccenic acid (11–18:1)**	*D. d.*, *P. p.*	Fruiting body, Plasmodium	[56,69,72]
**Dihomo-γ-linolenic acid (8,11,14–20:3)**	*A. c.*, *A. d.*, *A. n.*, *F. s.*, *L. e.*, *L. f.*, *P. sp.*, *T. f.*, *T. v.*	Fruiting body	[56,70,72]
**Docosahexaenoic Acid (DHA, 22:6)**	*A. c.*, *A. d.*, *A. n.*, *F. s.*, *L. e.*, *L. f.*, *P. sp.*, *T. f.*, *T. v.*	Fruiting body	[56,70]
**Docosadienoic acid (C_22_:2)**	*A. c.*, *A. d.*, *A. n.*, *F. s.*, *L. e.*, *L. f.*, *P. sp.*, *T. f.*, *T. v.*	Fruiting body	[56,70]
**Docosatetraenoic acid (C_22_:4)**	*L. t.*	Plasmodium	[56,74]
**Docosatrienoic acid (C_22_:3)**	*A. c.*, *A. d.*, *A. n.*, *F. s.*, *L. e.*, *L. f.*, *P. sp.*, *T. f.*, *T. v.*, *F. c.*, *L. t.*	Fruiting body	[56,74,75]
**Docosenoic acid (C_22_:1)**	*A. c.*, *A. d.*, *A. n.*, *F. s.*, *L. e.*, *L. f.*, *P. sp.*, *T. f.*, *T. v.*	Fruiting body	[56,70]
**Delta-5-fatty acid desaturase (enzyme activity)**	*D. d.*	Fruiting body	[65,66]
**Eicosapentaenoic acid (EPA)**	*A. c.*, *A. d.*, *A. n.*, *F. s.*, *L. e.*, *L. f.*, *P. sp.*, *T. f.*, *T. v.*	Fruiting body	[56,70]
**Erucic acid (13–22:1)**	*A. c.*, *A. d.*, *A. n.*, *F. s.*, *L. e.*, *L. f.*, *P. sp.*, *T. f.*, *T. v.*	Fruiting body	[56,70]
**Gadoleic Acid (9–20:1)**	*A. c.*, *A. d.*, *A. n.*, *F. s.*, *L. e.*, *L. f.*, *P. sp.*, *T. f.*, *T. v.*	Fruiting body	[56,70]
**Gondoic acid (cis-11-Eicosenoic acid)**	*A. d.*, *A. n.*, *F. s.*, *L. f.*, *P. sp.*	Fruiting body	[56,70,72]
**Heptadecenoic acid (C_17_:1)**	*A. c.*, *A. d.*, *A. n.*, *F. s.*, *L. e.*, *L. f.*, *P. sp.*, *T. f.*, *T. v.*	Fruiting body	[56,70]
**Hexadeca-4,7-dienoic acid**	*A. c.*, *A. d.*, *A. n.*, *F. s.*, *L. e.*, *L. f.*, *P. sp.*, *T. f.*, *T. v.*	Fruiting body	[30,56]
**Hexadecadienoic acid (C_16_:2)**	*A. c.*, *A. d.*, *A. n.*, *F. s.*, *L. e.*, *L. f.*, *P. sp.*	Fruiting body	[56,70]
**Hexadecenoic acid (C_16_:1,** Δ **)**	*A. c.*, *A. d.*, *A. n.*, *F. s.*, *L. e.*, *L. f.*, *P. sp.*, *T. f.*, *T. v.*	Fruiting body	[56,70]
**Linoleic acid (9,12–18:2)**	*A. c.*, *A. d.*, *A. n.*, *F. s.*, *L. e.*, *L. f.*, *P. sp.*, *T. f.*, *T. v.*	Fruiting body	[56,70,71,72,73]
**Mead acid (5,8,11–20:3)**	*A. c.*, *A. d.*, *A. n.*, *F. s.*, *L. e.*, *L. f.*, *P. sp.*, *T. f.*, *T. v.*	Fruiting body	[56,70]
**Myristoleic acid (9–14:1)**	*A. c.*, *A. d.*, *A. n.*, *F. s.*, *L. e.*, *L. f.*, *P. sp.*, *T. f.*, *T. v.*	Fruiting body	[56,70]
**Nervonic acid (C_24_:1,** Δ15 **)**	*A. c.*, *A. d.*, *A. n.*, *F. s.*, *L. e.*, *L. f.*, *P. sp.*	Fruiting body	[56,70]
**Octadeca-5,11-dienoic acid (5,11–18:2)**	*D. d.*, *Pol. p.*	Fruiting body	[56,72,73]
**Octadeca-6,9,12-trienoic acid**	*A. c.*, *A. d.*, *A. n.*, *F. s.*, *L. e.*, *L. f.*, *P. sp.*, *T. f.*, *T. v.*	Fruiting body	[56]
**Oleic acid (9–18:1)**	*A. c.*, *A. d.*, *A. n.*, *F. s.*, *L. e.*, *L. f.*, *P. sp.*, *T. f.*, *T. v.*, *D. d.*, *P. p.*	Fruiting body	[56,69,70,71,72]
**Palmitoleic acid (9–16:1)**	*D. d.*, *A. c.*, *A. d.*, *A. n.*, *F. s.*, *L. e.*, *L. f.*, *P. sp.*, *T. f.*, *T. v.*	Myxamoebae	[56,69,70,72]
**Paullinic acid (13–20:1)**	*A. c.*, *A. d.*, *A. n.*, *F. s.*, *L. e.*, *L. f.*, *P. sp.*, *T. f.*, *T. v.*	Fruiting body	[56,70]
**Sapienic acid**	*A. c.*, *A. d.*, *A. n.*, *F. s.*, *L. f.*, *P. sp.*, *T. f.*, *T. v.*	Fruiting body	[56,70]
**Stearidonic acid (SDA, 6,9,12,15–18:4)**	*A. c.*, *A. d.*, *A. n.*, *F. s.*, *L. e.*, *P. sp.*, *T. f.*, *T. v.*	Fruiting body	[56,70]
**Tetracosatetraenoic acid (C_24_:4)**	*D. d.*, *T. f.*, *T. v.*	Fruiting body	[56,69]
**Tri- and tetraenoic acids (general group)**	*T. f.*	Fruiting body	[70]

**Abbreviations:** A. c., *Arcyria cinerea*; A. d., *Arcyria denudata*; A. n., *Arcyria nutans*; D. d., *Dictyostelium discoideum*; F. c., *Fuligo cinerea*; F. s., *Fuligo septica*; L. e., *Lycogala epidendrum*; L. f., *Lycogala flavoscum*; L. t., *Lindbladia tubulina*; P. p., *Physarum polycephalum*; P. sp., *Physarum species*; Pol. p., *Polysphondylium pallidum*; T. f., *Trichia favogiena*; T. v., *Trichia varia*.

**Table 6:** Glycerophospholipids and related phospholipids reported in *Eumycetozoa*. Compounds are listed alphabetically, along with the species of origin, life-cycle structure, and relevant literature sources.

Compound Name	Source *Eumycetozoa* Species	Life-Cycle Structure	References
**1-(alk-1-enyl)-2-acyl-sn-glycero-3-phosphoethanolamine (plasmalogen PE)**	*A. sp.*, *F. s.*, *P. p.*, *T. v.*	Fruiting body	[56]
**1-alkyl-2-acetyl-sn-glycero-3-phosphocholine**	*A. sp.*, *F. s.*, *P. p.*, *T. v.*	Fruiting body	[56]
**1-alkyl-2-acyl-sn-glycero-3-phosphoethanolamine**	*A. sp.*, *F. s.*, *P. p.*, *T. v.*	Fruiting body	[56]
**1,2-Dipalmitoyl-sn-glycero-3-phosphoethanolamine**	*A. sp.*, *F. s.*, *P. p.*, *T. v.*	Fruiting body	[56]
**2-acyl-1-alkyl-sn-glycero-3-phosphocholine**	*D. d.*	Cellular stage, Plasmodium	[56,83]
**Cardiolipin**	*A. sp.*, *A. d.*, *A. f.*, *F. s.*, *P. p.*, *T. v.*, *T. c.*, *L. f.*	Fruiting body, Plasmodium	[56,77,78]
**Choline Lipids (general class)**	*A. sp.*, *F. s.*, *P. p.*, *T. v.*	Fruiting body	[56]
**Diacyl glycerophosphocholine**	*A. c.*, *A. d.*, *A. n.*, *F. s.*, *L. e.*, *L. f.*, *P. sp.*	Fruiting body	[56]
**Diacyl glycerophosphoethanolamine**	*T. f.*, *T. v.*, *D. d.*	Fruiting body	[56,69]
**Lysophosphatidylcholines**	*A. sp.*, *F. s.*, *P. p.*, *T. v.*	Fruiting body, Plasmodium, Myxamoebae	[56]
**Lysophosphatidylethanolamine**	*A. sp.*, *F. s.*, *P. p.*, *T. v.*	Fruiting body	[56,69]
**Phosphatidic acid**	*A. d.*, *P. c.*, *T. c.*, *L. f.*	Fruiting body, Plasmodium	[56,58,71,76]
**Phosphatidylethanolamine**	*A. sp.*, *F. s.*, *P. p.*, *T. v.*	Fruiting body, Plasmodium	[56,69,71]
**Phosphatidylcholine**	*A. sp.*, *F. s.*, *P. p.*, *T. v.*	Fruiting body	[56,69,71]
**Phosphatidylinositol**	*F. s.*, *P. p.*, *T. v.*	Fruiting body, Plasmodium	[56,71,75,79]
**Phosphatidylserine**	*A. sp.*, *F. s.*, *P. p.*, *T. v.*	Fruiting body	[56,78]
**Plasmenylcholine (1-O-alk-1’-enyl-2-acyl-sn-glycero-3-phosphocholine)**	*A. d.*, *L. f.*	Fruiting body, Plasmodium	[56,78]
**Plasmenylethanolamine (1-O-alk-1’-enyl-2-acyl-sn-glycero-3-phosphoethanolamine)**	*D. d.*	Cellular stage, Plasmodium	[56,80]
**Polycephalin B**	*F. s.*, *P. p.*	Plasmodium	[58,66,81,82]
**Polycephalin C**	*F. s.*, *P. p.*	Plasmodium	[58,66,81,82]

**Abbreviations:** A. c., *Arcyria cinerea*; A. d., *Arcyria denudata*; A. f., *Arcyria ferruginea*; A. n., *Arcyria nutans*; A. sp., *Arcyria spp.*; D. d., *Dictyostelium discoideum*; F. s., *Fuligo septica*; L. e., *Lycogala epidendrum*; L. f., *Lycogala flavoscum*; P. c., *Physarella corticifera*; P. p., *Physarum polycephalum*; P. sp., *Physarum* spp.; T. c., *Tubifera casparyi*; T. f., *Trichia favogiena*; T. v., *Trichia varia*.

**Table 7:** Sterols, steroids, and terpenoids reported in *Eumycetozoa*. Compounds are arranged alphabetically, with details on the source species, life-cycle stage, and key references.

Compound Name	Source *Eumycetozoa* Species	Life-Cycle Structure	References
**22-dihydroporiferasterol**	*P. p.*, *P. f.*	Myxoamoebae, Plasmodium	[82,84,86]
**24-Methylene-24-dihydrolanosterol (TMS derivative)**	*P. p.*	Plasmodium	[56,66,71]
**Beta-sitosterol**	*P. p.*	Plasmodium	[56,66,85]
**Brassicasterol**	*D. min.*	Fruiting body	[82]
**Campestanol**	*P. p.*	Plasmodium	[56,66,85]
**Campesterol**	*P. p.*	Plasmodium	[56,66,85]
**Cholesterol**	*P. p.*	Plasmodium	[56,66,85]
**Clionasterol**	*D. min.*, *D. s.*	Fruiting body	[66,82]
**Cytotoxic triterpenoid aldehyde lactone**	*T. d.*	Fruiting body	[56,79]
**δ-15-ergostenol**	*P. p.*, *P. f.*	Myxoamoebae, Plasmodium	[84,86]
**Ergostenol**	*P. p.*, *P. f.*	Myxoamoebae, Plasmodium	[82,84]
**Firmibasiol (geranylated bicyclogermacranol)**	*D. f.*	Fruiting body	[66,87]
**Glucoclionasterol**	*D. min.*	Fruiting body	[82]
**Lanosterol**	*P. p.*, *P. f.*	Plasmodium	[56,66,84]
**Lycoperdinoside A**	*T. d.*	Fruiting body	[89]
**Lycoperdinoside B**	*T. d.*	Fruiting body	[89]
**Mucoroidiol (protoilludane-type sesquiterpene)**	*D. m.*	Fruiting body	[66,87]
**Poliferasterol**	*D. min.*	Fruiting body	[82]
**Poriferastanol**	*P. p.*, *P. f.*	Myxoamoebae, Plasmodium	[84]
**Poriferasterol**	*P. p.*, *P. f.*	Myxoamoebae, Plasmodium	[82,84,86]
**Stigmasterol**	*P. p.*	Plasmodium	[56,66,85]
**Stigmastanol**	*P. p.*	Plasmodium	[56,66,85]
**Tubiferal A**	*T. d.*	Fruiting body	[58,66,79,88]
**Tubiferal B**	*T. d.*	Fruiting body	[58,66,79,88]

**Abbreviations:** D. f., *Dictyostelium firmibasis*; D. m., *Dictyostelium mucoroides*; D. min., *Didymium minus*; D. s., *Didymium sp.*; P. f., *Physarum flavicomun*; P. p., *Physarum polycephalum*; T. d., *Tubifera dimorphotheca*.

**Table 8:** Representative polyphenols from *Eumycetozoa*, listed alphabetically with species of origin, the developmental stage of isolation, and references.

Compound Name	Source *Eumycetozoa* Species	Life-Cycle Structure	References
**4-Methyl-5-pentylbenzene-1,3-diol (MPBD)**	*D. d.*	Fruiting body	[90,91,92]
**Dictyobiphenyl A**	*D. p.*	Fruiting body	[91,93]
**Dictyobiphenyl B**	*D. p.*	Fruiting body	[91,93]
**Dictyoterphenyl A**	*D. p.*	Fruiting body	[91,93]
**Dictyoterphenyl B**	*D. p.*	Fruiting body	[91,93]

**Abbreviations:** D. d., *Dictyostelium discoideum*; D. p., *Dictyostelium polycephalum*.

**Table 9:** Quinones and related polyketide metabolites isolated from *Eumycetozoa*. Compounds appear alphabetically, including any known structural variants (e.g., derivatives or dimethyl esters).

Compound Name	Source *Eumycetozoa* Species	Life-Cycle Structure	References
**2,3,5-Trihydroxynaphthoquinone**	*T. f.*	Fruiting body	[66,78]
**Chrysophysarin A**	*P. p.*, *L. e.*	Plasmodium, Fruiting body	[58,66,82,95]
**Dihydrolindbladione**	*L. t.*	Fruiting body, Plasmodium	[66,74,79]
**Homotrichione**	*D. b.*, *M. f.*, *M. v.*	Fruiting body	[66,82]
**Lindbladione**	*C. i.*, *L. t.*, *T. f.*, *D. i.*	Fruiting body, Plasmodium	[66,74,79,82]
**Lycogalic acid A**	*L. e.*	Fruiting body	[96,97]
**Lycogalic acid A derivatives**	*L. e.*	Fruiting body	[96]
**Lycogalic acid dimethyl ester A**	*L. e.*	Fruiting body	[96,98]
**Lycogalic Acid Dimethyl Ester B**	*L. e.*	Fruiting body	[96,98]
**Lycogalic Acid Dimethyl Ester C**	*L. e.*	Fruiting body	[96]
**Lycogarubin A**	*L. e.*	Fruiting body	[29,30,66,79,82]
**Lycogarubin B**	*L. e.*	Fruiting body	[29,30,66,82,97]
**Lycogarubin B analogues**	*L. e.*	Fruiting body	[66,97]
**Lycogarubin C**	*L. e.*	Fruiting body	[29,66,79,82,97]
**Lycogarubin C analogues**	*L. e.*	Fruiting body	[66,97]
**Physarorubinic acid A**	*P. p.*	Plasmodium	[56,58,66,82,94]
**Physarorubinic acid B**	*P. p.*	Plasmodium	[56,58,66,82,94]
**Trichione**	*A. d.*, *M. f.*, *T. sp.*	Fruiting body	[66,78,82]
**Vesparione**	*M. v.*	Fruiting body	[56,66,82,94]

**Abbreviations:** A. d., *Arcyria denudata*; C. i., *Cribraria intricata*; D. b., *Didymium bahiense*; D. i., *Didymium iridis*; L. e., *Lycogala epidendrum*; L. t., *Lindbladia tubulina*; M. f., *Metatrichia floriformis*; M. v., *Metatrichia vesparium*; P. p., *Physarum polycephalum*; T. f., *Trichia favogiena*; T. sp., *Trichia* sp.

**Table 10:** Other polyketides reported in *Eumycetozoa*. Compounds are listed alphabetically with their source species, life-cycle stage, and key references.

Compound Name	Source *Eumycetozoa* Species	Life-Cycle Structure	References
**6,7-Dimethoxydihydrolindbladione**	*L. t.*	Fruiting body	[66,79]
**6,7-Dimethoxylindbladione**	*L. t.*	Fruiting body	[79]
**6-Methoxydihydrolindbladione**	*P. c.*, *L. e.*, *L. t.*	Fruiting body	[30,66,79]
**7-Methoxylindbladione**	*L. t.*	Fruiting body	[66,79]
**Cribrarione A**	*C. p.*	Fruiting body, Plasmodium	[66,79,99]
**Cribrarione B**	*C. c.*	Fruiting body	[66,79,100]
**Cribrarione C**	*C. m.*	Fruiting body	[31,66]
**Desmethyl analogue of DIF-1 (dM-DIF-1)**	*D. d.*	Fruiting body	[90]
**Desmethyl-monochloro analogue of DIF-1 (Cl-THPH)**	*D. d.*	Fruiting body	[90]
**Monochasiol A, B, C, D, E, F, G, H**	*D. m.*	Fruiting body	[91,101]

**Abbreviations:** C. c., *Cribraria cancellata*; C. m., *Cribraria meylanii*; C. p., *Cribraria purpurea*; D. d., *Dictyostelium discoideum*; D. m., *Dictyostelium monochasioides*; L. e., *Lycogala epidendrum*; L. t., *Lindbladia tubulina*; P. c., *Perichaena chrysosperma*.

**Table 11:** Pigments identified in *Eumycetozoa*, including carotenoids, melanins, melanin-like substances, and other diverse coloration agents. Compounds are listed alphabetically, along with their source species, life-cycle structures, and pertinent references.

Compound Name	Source *Eumycetozoa* Species	Life-Cycle Structure	References
**Acidic carotenoids**	*D. d.*	Fruiting body	[102]
**Arcyriacyanin A**	*A. n.*, *A. d.*	Fruiting body	[58,66,78,111]
**Arcyriarubin A**	*L. e.*, *F. s.*, *A. d.*	Fruiting body, Plasmodium	[30,58,66,78,79,96,97]
**Arcyriarubin B**	*A. d.*, *F. s.*	Fruiting body, Plasmodium	[58,66,77,78,82]
**Arcyriarubin C**	*A. d.*, *A. f.*, *F. s.*	Fruiting body, Plasmodium	[58,66,77,78,79,82,105]
**Dihydroarcyriacyanin A**	*A. o.*, *A. f.*, *A. d.*	Fruiting body	[58,66,78,103,104]
**Dihydroarcyriarubin B**	*A. d.*, *A. f.*	Fruiting body	[66,78,105]
**Dihydroarcyriarubin C**	*A. f.*, *T. c.*	Fruiting body	[58,79,105]
**Dihydroarcyrioxocin A**	*A. d.*, *T. c.*	Fruiting body	[58,66,78,104,105]
**Fuligorubin A**	*F. s.*, *F. s.*, *P. p.*	Plasmodium	[56,58,66,78,79,82,95,106,107,108,109]
**Melanin**	*P. n.*	Fruiting body	[66]
**Melanin-like pigments**	*F. s.*	Sclerotium	[110]
**Naphthoquinone pigments**	*C. p.*	Fruiting body	[78,79]
**Physarochrome A**	*T. v.*	Fruiting body	[56,58,66,78,79,94]
**Zeta-carotene derivatives**	*D. d.*	Fruiting body	[66,102]

**Abbreviations:** A. d., *Arcyria denudata*; A. f., *Arcyria ferruginea*; A. n., *Arcyria nutans*; A. o., *Arcyria obvelata*; C. p., *Cribraria purpurea*; D. d., *Dictyostelium discoideum*; F. s., *Fuligo septica*; L. e., *Lycogala epidendrum*; P. n., *Physarum nebulosum*; P. p., *Physarum polycephalum*; T. c., *Tubifera casparyi*; T. v., *Trichia varia*.

**Table 15:** A summary of selected bioactive compounds identified in true slime molds (*Eumycetozoa*) with at least potential application in sustainable pest or pathogen control.

Category	Compound(s)	Key Bioactivity	Potential Role in Pest/Pathogen Control
**Carbohydrates, Their Derivatives, and Glycoconjugates**			
	**Lycogalinosides A, B**	Inhibitory activity against Gram-positive bacteria [55,56,57,58]	Antibacterial agents against Gram-positive pathogens
**Proteinogenic and Non-Proteinogenic Amino Acids**			
	**Alanine, Aspartic Acid, Threonine, Proline, Leucine, Lysine, Glutamic Acid**	Spore germination and cyst formation [59,60]	Development of amino acid analogs or nutrient-limiting strategies to disrupt spore germination in pathogens
**Enzymes, Peptides, and Macromolecular Proteins**			
	**Hemagglutinin (physarumin), Discoidin**	Binds cell-surface carbohydrates (hemagglutination, adhesion) [67]	Selective interference with insect or microbial cell-surface interactions via lectin-based approaches
	**Glorin**	Chemotactic signal guiding aggregation [68]	Attractants or repellents
**Sterols, Steroids, and Terpenoids**			
	**Tubiferal A, Tubiferal B**	Cytotoxic and multidrug-resistance-reversing effects [79,88]	Enhancing efficacy of existing antimicrobials
	**Lycoperdinosides A, B**	Antibacterial and cytotoxic [89]	Controlling phytopathogens or insect pests through disruption of microbial and cellular processes
**Polyphenols**			
	**4-methyl-5-pentylbenzene-1,3-diol (MPBD)**	Antimicrobial; induces stalk differentiation [90,92]	Bio-based agent for controlling plant-pathogenic microbes
**Quinones**			
	**2,3,5-Trihydroxynaphthoquinone, Homotrichione, Trichione, Vesparione**	Antimicrobial and cytotoxic [78,82]	Controlling bacterial or fungal pathogens
	**Cribrarione A, B, C; Lindbladione, Dihydrolindbladione**	Antibacterial activities [74,82]	Targeting plant-associated bacteria.
**Other Polyketides**			
	**Lindbladione Derivatives**	Antimicrobial and cytotoxic [79]	Bio-based bactericides or fungicides
	**Cribrariones A, B, C**	Antimicrobial activity [99]	Controlling bacterial or fungal pathogens
**Pigments**			
	**Carotenoids (e.g., zeta-carotenes)**	Photoprotective [66]	Enhancing UV tolerance in beneficial biocontrol agents
	**Bisindole Pigments**	Cytotoxic or cell-cycle inhibitory [103]	Impacting eukaryotic pathogens susceptible to cytotoxic mechanismss
	**Melanin and Melanin-Like Pigments**	Photoprotective and antioxidant [110]	Protective coatings (e.g., seed treatments) to reduce oxidative stress
	**Naphthoquinones**	Fungicidal/bactericidal in other organisms [79]	New antifungal or antibacterial agents
**Alkaloids and Indole Derivatives**			
	**Staurosporine Derivatives**	Protein kinase inhibitors, cytotoxic [76,79]	Novel biofungicides or bactericides via disruption of pathogen signaling
	**Arcyriaflavins (A–C)**	Antibiotic and cytotoxic [78,97]	Targeting plant-associated bacteria or spoilage microbes
	**Makaluvamines (A, B, I)**	Antibacterial, topoisomerase inhibition [29,82]	Limiting bacterial growth
**Polyenes and Polyacetylenes**			
	**Ceratiopyrons A–D (Ceratioflavin A)**	Antibiotic and cytotoxic [66,82]	Developing new antibacterial agents
	**Cinereapyrroles A, B**	Antibiotic [79,97]	Controlling bacterial or fungal pathogens
	**Dictyopyrones, Dihydrodictyopyrones**	Inhibit cell proliferation and modulate morphological development (e.g., stalk cell differentiation [116,117]	Disrupting developmental processes or spore formation in pests/pathogens via interference with cellular growth and differentiation pathways
	**Bispyrone Analogs (Dictyomedins)**	Delay or inhibit morphological differentiation in *Dictyostelium* [118,119]	Disrupting development (e.g., aggregation, spore formation) in pests or pathogens by interfering with key differentiation processes
**Other Eumycetozoa-Specific Secondary Metabolites**			
	**Acyltetramic Acids**	Antimicrobial [66,78]	Controlling bacterial or fungal pathogens
	**Melleumin A, B**	Antimicrobial and cytotoxic [79,123]	Targeted bacterial suppression
	**D-1 (heterocyclic antibiotic fraction)**	Antibiotic activity [32,33]	Controlling bacterial or fungal pathogens
	**Enteridinines A, B**	Inhibitory effects on bacterial growth [66,125]	Controlling bacterial pathogens

## Data Availability

Data are contained within the article.
